# Amino acid metabolic signaling influences *Aedes aegypti* midgut microbiome variability

**DOI:** 10.1371/journal.pntd.0005677

**Published:** 2017-07-28

**Authors:** Sarah M. Short, Emmanuel F. Mongodin, Hannah J. MacLeod, Octavio A. C. Talyuli, George Dimopoulos

**Affiliations:** 1 W. Harry Feinstone Department of Molecular Microbiology and Immunology, Bloomberg School of Public Health, Johns Hopkins University, Baltimore, Maryland, United States of America; 2 Institute for Genome Sciences, University of Maryland School of Medicine, Baltimore, Maryland, United States of America; Colorado State University, UNITED STATES

## Abstract

The mosquito midgut microbiota has been shown to influence vector competence for multiple human pathogens. The microbiota is highly variable in the field, and the sources of this variability are not well understood, which limits our ability to understand or predict its effects on pathogen transmission. In this work, we report significant variation in female adult midgut bacterial load between strains of *A*. *aegypti* which vary in their susceptibility to dengue virus. Composition of the midgut microbiome was similar overall between the strains, with 81–92% of reads coming from the same five bacterial families, though we did detect differences in the presence of some bacterial families including *Flavobacteriaceae* and *Entobacteriaceae*. We conducted transcriptomic analysis on the two mosquito strains that showed the greatest difference in bacterial load, and found that they differ in transcript abundance of many genes implicated in amino acid metabolism, in particular the branched chain amino acid degradation pathway. We then silenced this pathway by targeting multiple genes using RNA interference, which resulted in strain-specific bacterial proliferation, thereby eliminating the difference in midgut bacterial load between the strains. This suggests that the branched chain amino acid (BCAA) degradation pathway controls midgut bacterial load, though the mechanism underlying this remains unclear. Overall, our results indicate that amino acid metabolism can act to influence the midgut microbiota. Moreover, they suggest that genetic or physiological variation in BCAA degradation pathway activity may in part explain midgut microbiota variation in the field.

## Author summary

The mosquito midgut microbiota plays an important role in mosquito susceptibility to human pathogens and therefore is an important component of mosquito disease transmission. The microbiota can be highly variable, however, and the sources of this variation are not well understood. In this work, we aimed to improve our understanding of the ways in which the mosquito can influence its microbiota. To do this, we utilized strains of *Aedes aegypti* that vary in their midgut microbial load. We compared the transcriptomes of two strains with highly disparate bacterial loads, and found that genes involved in amino acid metabolism (specifically branched chain amino acid degradation) were differentially regulated between the strains. We found that when we silenced these genes using an RNA interference approach, bacterial loads increased in a strain-specific manner, confirming a role for these genes in controlling proliferation of the midgut microbiota. Overall, these results suggest that differences in amino acid metabolism or catabolism in the mosquito could have important implications for the mosquito microbiota.

## Introduction

*Aedes aegypti* mosquitoes are a primary vector for multiple arboviruses that infect humans including dengue virus, chikungunya virus, yellow fever virus and Zika virus. Dengue alone inflicts a staggering disease burden, with approximately 3.9 billion people globally at risk of contracting the virus [1] and an estimated prevalence of 390 million cases per year [2]. Because of their central role in pathogen transmission, understanding vector competence of *Aedes* mosquitoes is critically relevant to developing new methods of reducing this disease burden and improving public health. One issue complicating our ability to understand and/or predict vector competence is the fact that it varies substantially in natural populations [3]. This variation has been attributed in part to both genetic heterogeneity as well as environmental factors such as temperature [4–7]. One additional factor that has the potential to influence variation in vector competence is the mosquito midgut microbiota [8].

Previous work has shown that reducing the number of bacteria in the mosquito midgut through antibiotic ingestion results in increased dengue virus titers in *A*. *aegypti* [9]. Additionally, experimental introduction of multiple bacterial species into the mosquito midgut results in decreased dengue titers [10,11], while at least one species of bacteria, *Serratia odorifera*, has been shown to increase dengue and chikungunya susceptibility when present in the midgut [12,13]. These findings demonstrate how the load and species composition of the midgut microbiota can influence vector competence. They also suggest that variability in either factor could contribute to differences in vector competence and pathogen transmission in natural populations [8], and thereby emphasize the importance of understanding the nature and causes of midgut microbiota variability.

In natural mosquito populations, the composition of the midgut and whole-body microbiota has been repeatedly reported to vary between sampling locations [e.g. 14–16]. This spatial variation is hypothesized to be largely attributable to differences in the local microbial fauna [15,17], given that both larvae and adults ingest bacteria from the environment. Larvae ingest bacteria from the breeding water, which can be transstadially transmitted to the adult stage [17,18]. Adults have been shown to ingest bacteria from larval breeding water [19] and are also thought to acquire microbes from flower nectar, which contains bacteria [20]. Others have shown, however, that individuals of the same species collected from the same field site or from the same laboratory population over time also display substantial variation in midgut microbial load and composition [21–23], suggesting that microhabitat, physiology and life history could also affect midgut microbiota variation. Additionally, it has been reported that microbiota composition can vary between mosquito species [21,24], and that composition and total bacterial load varies between strains of the same species, [25], suggesting that genetic variation may also influence midgut bacterial colonization and proliferation. Given the substantial variation observed in the midgut microbiota, a thorough understanding of how the microbiota influences vector competence and vectorial capacity requires improved knowledge of the basic biology of the microbiota itself.

Current research suggests that factors such as metamorphosis, feeding (both on sucrose and blood), and immune system signaling all have the capacity to influence the bacterial species composition and total number of bacteria in the mosquito midgut. During metamorphosis, midgut bacterial loads are dramatically reduced [26], though there is evidence that some bacteria persist through the pupal stage into adulthood via transstadial transmission [17,26]. Sugar feeding alters the composition of the midgut microbiota, resulting in a decrease in microbial diversity [23]. Blood feeding causes dramatic proliferation of bacteria in the midgut [27–29] coupled with substantial decreases in population diversity [23], possibly because certain bacterial species are better able to utilize the blood meal as a nutrient source. The increase in bacterial load after blood feeding has been shown in *Anopheles gambiae* to be facilitated by a peroxidase/dual oxidase system in the midgut which promotes formation of a crosslinked layer of proteins in the midgut lumen immediately after blood feeding [28]. This layer prevents the midgut epithelium from coming into contact with microbes and therefore prevents their detection and elimination by the immune system [28]. In addition to this physical barrier, bacterial proliferation after a blood meal has been shown in *A*. *aegypti* to be promoted by heme in the blood, which serves to decrease anti-bacterial reactive oxygen species (ROS) in the midgut, allowing bacteria to proliferate [27]. The mosquito’s immune system, specifically the IMD pathway, also influences bacterial load in the midgut. Silencing the IMD upstream receptor PGRP-LC as well as the downstream transcription factor Rel-2 leads to increased midgut bacterial loads [22,30]. Additionally, activating the pathway through overexpression of Rel-2 or silencing the negative regulator Caudal leads to decreased midgut bacterial loads [31,32]. Collectively, this work has successfully identified multiple factors that act to regulate the midgut microbiota. It remains unclear, however, whether any of these pathways influence the aforementioned intra-species variability in the midgut microbiota.

Here we focused on determining factors that may contribute to differences in midgut bacterial load between adult females from multiple strains of the same mosquito species, *A*. *aegypti*. We first investigated whether mosquitoes from five strains of *A*. *aegypti* [Rockefeller (Rock), Singapore (Sing), Orlando, Waco and Bangkok] reared in highly similar bacterial environments as larvae varied in midgut microbial load and/or composition as adults. We found significant variation in midgut microbial load between these strains. We also found that midgut bacterial community composition was largely the same between strains, though there were some notable differences. We then proceeded to investigate potential mechanisms underlying the variation in midgut bacterial load between two strains that were maximally disparate. Using whole genome transcriptome analysis, we compared midgut mRNA abundance between the two strains and found that many genes involved in metabolism were differentially regulated. One pathway that was highly represented among differentially expressed genes was the valine-leucine-isoleucine degradation pathway. Silencing of multiple genes in this pathway each resulted in a significant increase in bacterial load in one mosquito strain, and a consequent elimination of the difference in bacterial load between the strains. Taken together, these data suggest that differences in midgut bacterial load may be influenced by variation in amino acid metabolism in the midgut.

## Methods

### Ethics statement

This study was carried out in strict accordance with the recommendations in the Guide for the Care and Use of Laboratory Animals of the National Institutes of Health. Mice were used only for mosquito rearing as a blood source, according to the approved protocol. The protocol was approved by the Animal Care and Use Committee of the Johns Hopkins University (Permit Number: M006H300).

### Strain information and maintenance

Strains of *Aedes aegypti* that were used in the current work are described in detail in [33]. In brief, the Rockefeller (Rock) strain was isolated from the Caribbean in the 1930s [34], the Orlando strain was isolated from Orlando, Florida USA prior to 1940 [35], the Waco strain was isolated from Waco, Texas USA in ~1987 [36], the Bangkok strain was isolated from Bangkok, Thailand in 2011 [33] and the Singapore (Sing) strain was isolated from Singapore, Singapore in 2010 [33]. All strains were maintained at 27°C and 80% humidity with a 14:10 hour light:dark cycle. As larvae, they were provided larval food (liver powder, tropical fish flake food, and rabbit food pellets mixed in a 2:1:1 ratio) *ad libitum* and upon eclosion were then transferred to sterile cages and provided with 10% sucrose *ad libitum*.

### Mosquito rearing for experiments

For all experiments, mosquitoes were maintained at 27°C and 80% humidity with a 14:10 hour light:dark cycle. Unless otherwise stated, eggs were hatched in sterile water in a vacuum and then moved to larval rearing pans containing 1L sterile water and provided larval food *ad libitum*. At 3 days post hatching, larvae from all strains were removed to separate sterile beakers, and as much breeding water as possible was removed from the beaker. An equal volume of rearing water from each strain was then pooled by passing through a coarse filter (Whatman #1 filter paper) into a common beaker. 200ml of this pooled water plus 1L sterile water was added to fresh trays and larvae were returned to these pans and fresh food was added. This was repeated without filtering on day 5 post hatching. On day 6–7 post hatching, water was mixed again between strains and portioned into pupal cups. Pupae plus some L4 larvae from each strain were added to these cups containing the pooled water and allowed to eclose in separate cages over a period of 2 days. Adults were provided 3% sterile sucrose *ad libitum*.

### Blood feeding

3–6 days post eclosion, females from each strain were provided a blood meal via artificial membrane feeding system. Females were starved overnight to encourage feeding. The blood meal contained 40% human defibrinated red blood cells, 10% human heat-inactivated serum, 40% 1X Phosphate Buffered Saline (PBS) and 10% 10mM ATP to facilitate feeding. In experiments where bacteria were introduced via blood meal, the 1X PBS was replaced with bacterial liquid culture resuspended in 1X PBS. Females were allowed to feed for 30–60 minutes and were then cold-anaesthetized and those that had not fed were removed. Non-blood fed controls were also cold-anaesthetized at this time to control for effects of cold shock. All females were then returned to the incubator to recover and given 3% sterile sucrose *ad libitum*.

### Midgut dissection

Females to be dissected were cold-anaesthetized and surface sterilized in 70% ethanol for 1 minute followed by 2 washes in 1X PBS. Midguts were dissected on a sterile glass slide on a cold block using sterile forceps that were cleaned between each dissection with 70% EtOH. Unless samples were to be pooled, each individual mosquito was dissected in a separate pool of 1X PBS. If bacteria were to be cultured or DNA extracted from the midgut(s), samples were transferred to a 1.5 mL microcentrifuge tube containing 1X PBS. If RNA and DNA were to be extracted from the sample or pool of samples, midgut(s) were moved to a 1.5 mL microcentrifuge tube containing TRIZOL reagent and kept on ice until freezing at -80C.

### Culturing and quantifying bacteria

Midguts were homogenized in 1X PBS using a sterile pestle. For the initial experiment using all five strains, the homogenate was then diluted 1:100 in additional sterile 1X PBS and 80 μL of the undiluted and diluted homogenates were then spread on LB agar plates. For Singapore blood fed individuals in this experiment, bacteria were too numerous for many samples to be accurately counted. We therefore assigned the maximum value we obtained from countable samples in that treatment to uncountable plates as a conservative under-estimate of total bacterial number. For the subsequent time course experiment using only Rock and Sing strains, the homogenate was diluted 1:100 and 1:10^4^ and 50 μL of the undiluted and diluted homogenates were then spread on LB agar plates. For larval water, an aliquot of breeding water was taken from rearing pans at the 4^th^ larval instar and pupal stage and serially diluted in sterile LB. 80μL of each dilution was plated on LB agar. For all experiments, plates were incubated at room temperature for 48 hours. Colony forming units (CFUs) were counted by hand when there was a high diversity of colony types and by a colony counter (aCOLyte) when fewer colony types were detected.

### Colony isolation and identification

Bacterial colony types were first characterized for each experiment based on margin, form, elevation, color and translucency. Each colony was then re-isolated on LB agar and single colonies were used to generate liquid cultures. Sequencing was generally performed using single bacterial colonies as a template. In cases where direct colony PCR was unsuccessful, gDNA was extracted from liquid culture and used as template. In rare cases, we were unable to re-isolate colonies that grew and in those cases sequencing was performed using the original colony as template. The 16S rDNA gene was sequenced for each colony type using the 27F and 1492R universal 16S primers (S1 Table). We then used Sequence Match through the Ribosomal Database Project (http://rdp.cme.msu.edu/, [37]) to identify sequences to genus level, considering a match to be at or above 97% similarity. Though we classified to genus level, we present these data at family level to allow for comparison with 16S high-throughput sequencing data. Genus level data can be found in S1 File. All families but one (Enterobacteriaceae) were represented by a single genus, therefore diversity at the family level closely mirrors that at the genus level.

### DNA/RNA extraction and cDNA synthesis

To extract DNA and RNA from the same midgut or pool of midguts, we homogenized midgut(s) in TRIzol reagent (Invitrogen) using a sterile pestle and followed the manufacturer’s instructions. RNA was resuspended in 30uL DEPC H_2_O and DNA was resuspended in 30uL UV-treated DEPC H_2_O. To extract only DNA, we performed a standard phenol:chloroform extraction [38], with the addition of an initial step where lysozyme was added at a final concentration of 20mg/ml and the samples were incubated at 37°C for 1 hour. gDNA was removed from RNA samples using the TURBO DNA-*free* Kit according to the manufacturer’s instructions. 500ng RNA was primed with Oligo(dT) and M-MLV Reverse Transcriptase (Promega) was used to synthesize cDNA according to the manufacturer’s instructions.

### Microarray sample collection, experimental design, labeling, hybridization and feature extraction

Rockefeller and Singapore mosquitoes were reared in parallel and provided 3% sucrose + antibiotics [penicillin (100U/ml), streptomycin (100ug/ml), and gentamycin (75ug/ml)] upon eclosion. Because adults were to be treated with antibiotics, larval water was not mixed during rearing. Five to seven days post eclosion, females from each strain were randomly allocated to one of three treatments: (1) maintenance on 3% sucrose, (2) sterile blood meal or (3) blood meal containing bacterial cocktail. Blood meals contained 40% human defibrinated red blood cells, 10% human heat-inactivated serum, 10% 10mM ATP and 40% 1X PBS (for the sterile blood meal) or 40% bacterial cocktail (for the septic blood meal). To create the bacterial cocktail, seven bacterial species were grown overnight in liquid bacterial culture and each culture was washed 2X in 1X PBS and then standardized to an optical density of 1.0 (±0.1) at 600nm. An equal volume of each bacterial culture was then combined to create a cocktail. The bacterial species used are detailed in S2 Table. Approximately 12 hours after blood feeding, 15 midguts from all six strain/treatment combinations were dissected in sterile 1X PBS and transferred immediately to TRIzol reagent. Samples were stored at -80°C until RNA extraction, which was performed according to the manufacturer’s instructions. Genomic DNA was removed from all samples using TURBO DNA-free (Invitrogen) according to the manufacturer’s instructions and all samples were analyzed on the Agilent 2100 Bioanalyzer (Agilent Technologies Inc., Santa Clara, CA) to verify the integrity of the RNA. All samples were labeled using the Two-Color Low Input Quick Amp Labeling Kit (Agilent Technologies) according to the Two-Color Microarray-Based Gene Expression Analysis Protocol (Agilent Technologies). 200ng of RNA from each sample was used as input for the labeling reaction, and experimental samples were labeled with Cy5 fluorescent dye while reference samples were labeled with Cy3. Labeled cRNA was purified using RNeasy columns (Qiagen). All samples were co-hybridized to the same reference sample, which was formed by pooling an equal amount of RNA from all samples collected in the experiment. All six samples from the same biological replicate were hybridized to six arrays on the same slide, and a different slide was used for each replicate, resulting in a complete block design. Hybridization was performed according to Agilent’s Two-Color Microarray-Based Gene Expression Analysis Protocol. Feature extraction was performed using an Agilent Scanner and Agilent Feature Extraction Software.

### Microarray data analysis

Differential expression analysis was performed using the package limma in R [39]. Background correction was performed for all arrays using the normexp method [40]. Signals were normalized first within array using loess within-array normalization [41] and then between arrays using quantile normalization. Replicate probes for each gene were then averaged and differential expression for each gene was determined. The following contrasts were performed: Rock sucrose vs. Rock blood fed; Sing sucrose vs. Sing blood fed; Rock blood fed. vs. Rock bacteria fed; Sing blood fed vs. Sing bacteria fed. Two additional contrasts were performed to determine whether any genes responded to blood feeding or bacterial feeding significantly differently between strains (*e*.*g*. *=* (Rock sucrose–Rock bl.f.)–(Sing sucrose–Sing bl.f.)). P-values were adjusted for multiple comparisons using the Benjamini-Hochberg method. Gene lists from each pairwise comparison were further refined to include genes only affected by treatment in a single strain (i.e. strain-specific differential expression). Genes showing strain-specific differential expression were then used for Gene Ontology and KEGG analyses. Gene Ontology analysis was performed for biological process GO terms using the GOstats package in R [42] and p-values of enriched terms were adjusted using the Benjamini-Hochberg method. Redundancy in lists of significant GO terms was reduced using the online tool REVIGO [43]. KEGG pathway analysis was performed using DAVID [44,45].

### 16S rDNA community profiling

Sample prep and sequencing: For 16S rDNA high-throughput sequencing, we characterized a subset of samples used in the single time point bacterial load analysis. Upon midgut collection and homogenization, all remaining homogenate was preserved at -80°C in TRIzol (Invitrogen) and DNA was extracted using TRIzol according to the manufacturer’s instructions. We then pooled an equal volume of DNA from all samples of Rockefeller-sucrose, Rockefeller-blood fed, Singapore-sucrose and Singapore-blood fed treatments from a single experimental replicate (Rock sucrose, n = 8; Sing sucrose, n = 8; Rock blood fed, n = 15; Sing blood fed, n = 10). 16S rRNA gene sequencing was performed using Illumina MiSeq as previously described [46]. Briefly, the universal primers 319F and 806R were used to PCR amplify the V3V4 16S hypervariable regions [47] in 96-well microtiter plates using procedures previously published [46,48,49]. Negative controls without a template were processed for each primer pair. The presence of amplicons was confirmed using gel electrophoresis, after which the SequalPrep Normalization Plate kit (Life Technologies) was used for clean-up and normalization (25 ng of 16S PCR amplicon pooled for each sample) before sequencing. 16S rRNA reads were initially screened for low quality bases and short read lengths [46]. Paired-end read pairs were then assembled using PANDAseq [50] and the resulting consensus sequences were de-multiplexed (i.e. assigned to their original sample), trimmed of artificial barcodes, 5’ primer and 3’ primers and assessed for chimeras using UCHIME in *de novo* mode [51] implemented in QIIME [52] (S1 Data and S2 Data). OTU picking was performed in QIIME using a 97% similarity cutoff and taxonomic assignments were made using the GreenGenes 16S sequence database (version 13.8) [52,53]. Weighted UniFrac values, Nonmetric Multidimensional Scaling (NMDS) ordination and accompanying heatmap were generated in R using the package Phyloseq (www.r-project.org). Weighted UniFrac is a distance metric incorporating both the phylogenetic distance between the OTUs found in each sample as well as the difference in abundance of each OTU between samples [54]. We chose to use this metric rather than the qualitative unweighted UniFrac (which does not incorporate abundance data [55]) because we were particularly interested in identifying differences in composition that could account for the differences we detected in bacterial load. We normalized the weighted UniFrac values, such that a value of 0 indicates identical bacterial composition between samples and a value of 1 indicates no overlap.

### RNAi silencing

Annotation information for genes selected for RNAi-based silencing assays was verified by BLASTN using the NCBI non-redundant nucleotide collection as the search set. For additional validation, we performed a reverse BLASTN, using the human ortholog of each gene to BLAST the *Aedes aegypti* genome. In each case, the top hit was our gene of interest further confirming the annotation of these genes. PCR products to be used for dsRNA synthesis were generated using the T7 primers in S1 Table. PCR products were run on a 1% agarose gel and gel-purified if more than one product was present. Cleaned-up PCR products were then Sanger sequenced to verify the PCR product sequence matched that of the target gene. dsRNA was synthesized using the HiScribe T7 Quick High Yield RNA Synthesis Kit (New England Biolabs) according to the manufacturer’s instructions. dsRNA was run on a 1% agarose gel after synthesis and purification to verify that a single dsRNA product of the appropriate size was produced. Three to five days post eclosion, Rockefeller and Singapore females were cold-anaesthetized and injected in the thorax with 200ng dsRNA targeting a single gene. Injections were performed using a Nanoject II Auto-Nanoliter Injector (Drummond Scientific). dsRNA targeting the eGFP gene was injected into a separate group from each strain to serve as an injection control. After injection, females were returned to the incubator to recover and provided 3% sucrose *ad libitum*. Midguts were dissected from all treatments and strains approximately 48 hours after injection. Midguts were stored in TRIzol at -80°C until RNA/DNA extraction.

### Dengue infections and plaque assays

Dengue virus propagation, mosquito infection, and plaque assays were conducted as described previously [10,56]. In brief, to propagate the virus, C6/36 cells were grown in MEM medium (Gibco, USA) supplemented with 10% heat-inactivated FBS, 1% non-essential amino acid solution, 1% L-glutamine, and 1% penicillin-streptomycin to 80% confluency, infected with dengue virus serotype 2, and the infected cells were then incubated for five days at 32°C and 5% CO_2_. Virus was harvested from cell culture supernatant as well as from lysed cells subjected to three freeze-thaw cycles and stored at -80°C until used for infection. Rockefeller strain females were injected with dsRNA targeting AAEL003125, AALE004137, AAEL006928, or eGFP as described above. Approximately 48 hours after dsRNA injection, females were provided a blood meal containing virus stock mixed 1:1 with commercial human blood and supplemented with 10% heat inactivated serum and 1% 100mM ATP. Midguts were dissected as described above from blood fed adult females after seven days and stored at -80°C in 150ul DMEM media until used for viral titration. DMEM was supplemented with 10% heat-inactivated FBS, 1% L-glutamine, 1% penicillin-streptomycin, and 5μg/ml Plasmocin. Titration was performed via plaque assay in BHK-21 cells; cells were fixed five days after infection with each sample and plaques were visualized by staining fixed cells with 1% crystal violet.

### Quantitative real-time PCR

Quantitative real-time PCR was performed using the StepOnePlus Real-Time PCR System (Applied Biosystems) and SYBR Green PCR Master Mix (ThermoFisher Scientific). To test silencing by RNAi, cDNA synthesized from RNA was used as template; to quantify bacterial 16S levels, gDNA was used as template. All reactions were performed in duplicate. Primers used for qPCR can be found in S1 Table.

### Amino acid analysis

Rockefeller and Singapore strain female midguts were dissected at 5–7 days post-eclosion and total valine, leucine, and isoleucine levels were assayed via Amino Acid Analysis by the Molecular Structure Facility in the Proteomics Core, University of California, Davis. Twenty-five midguts were collected from each strain and pooled in 50μL 1X PBS, and the entire experiment was repeated three times. All samples were homogenized using a sterile pestle and hydrolyzed using 6N HCl and 1% phenol for 24 hours at 110°C under a vacuum. Samples were dissolved in sodium diluent (Pickering) supplemented with 40nmol/mL NorLeucine as an internal standard and analyzed using an L-8800 Hitachi amino acid analyzer. This system utilizes an ion-exchange column (Transgenomic) for separation followed by a secondary reaction with ninhydrin for amino acid detection. Standardization corrections and calculations to determine amino acid molar fraction were performed by the UC Davis Molecular Structure Facility.

### Statistical analysis

Zero-inflated data analysis was performed using the hurdle function in the pscl package in R as recommended by Zuur *et al*. [57]. This is a two-step analysis, the first being a regression on count data from all samples with nonzero values (here on referred to as “count data”). In our case, this was a negative binomial regression due to overdispersion of the count data. The second step is a binomial regression with the binary response variable representing presence or absence of bacteria in the gut (here on referred to as “presence/absence data”). For the single time point bacterial load analysis, we started with a model that included strain, feeding status and a two-way interaction between these variables. For the multiple time point bacterial load analysis, we started with a model that included strain, feeding status and time post blood feeding, as well as a three-way interaction between these factors and two-way interactions between each pairwise combination of factors. We then performed stepwise backwards selection on each model to determine the best model as well as the significance of relevant terms. ANOVA, Tukey’s HSD, and Dunnett’s tests were performed in R. Raw data for all figures can be found in S1 File.

## Results and discussion

### Midgut bacterial load varies significantly between *Aedes aegypti* strains

**Fig 1:**
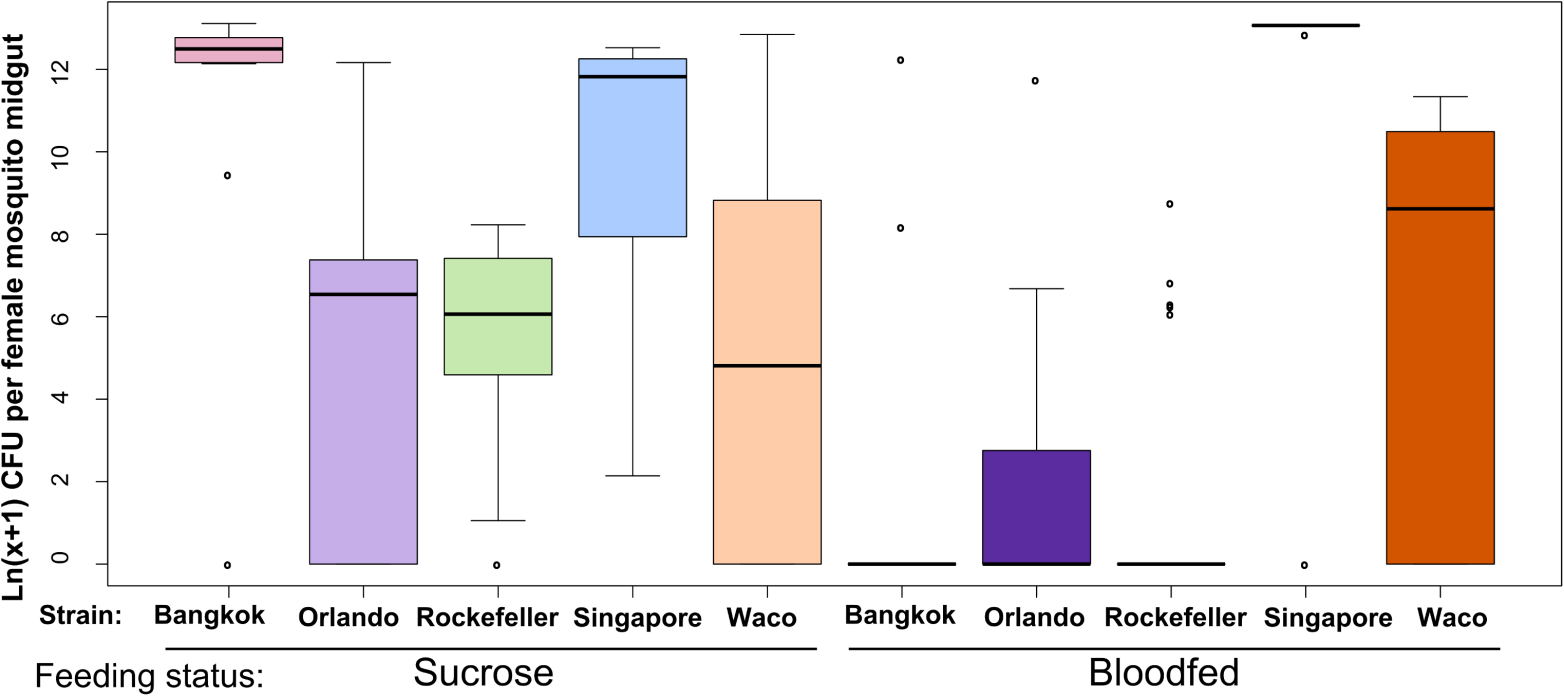
*A*. *aegypti* strains vary significantly in their LB-cultivable gut microbial load. We reared female *A*. *aegypti* from five strains in parallel and mixed the breeding water between strains three times during larval/pupal development. We maintained adults on 3% sucrose until 5 days post eclosion, at which point a subset of the females were given a sterile blood meal. At 48 hours post blood feeding, we externally sterilized all adults with 70% EtOH, dissected midguts from each strain/feeding treatment, homogenized individual midguts in 1X PBS using a sterile pestle and diluted the homogenate 1:100 using additional 1X PBS. We spread 80ul of undiluted and 1:100 diluted homogenate on LB agar plates and allowed the plates to grow for 48 hours at room temperature. We combined counts from all bacterial genera found in each individual to determine total CFUs per individual female midgut. We collected data over two experimental replicates; sample sizes are as follows: Bkk_sucrose_: n = 13, Bkk_blood_: n = 11, Orl_sucrose_: n = 18, Orl_blood_: n = 24, Rock_sucrose_: n = 18, Rock_blood_: n = 25, Sing_sucrose_: n = 13, Sing_blood_: n = 11, Waco_sucrose_: n = 7, Waco_blood_: n = 8. For many individuals, zero bacterial colonies grew on the LB plates, which resulted in this dataset being zero-inflated. We therefore performed a zero-inflated data analysis to test for the effect of strain, feeding status and a strain × feeding status interaction on the presence/absence of bacteria or total bacterial load. We then performed stepwise backwards selection of model terms using likelihood ratio tests to assess the significance of each dropped term. For count data (*i*.*e*. the part of the model analyzing individuals with at least one CFU), strain was a significant predictor of bacterial load (p = 6.19 x 10^−14^) and we detected no interaction between strain and feeding status. For presence/absence data (*i*.*e*. the part of the model analyzing the presence or absence of bacteria), we detected a significant interaction (p = 0.01739) between strain and feeding status.

While a role for environment has been implicated in shaping the mosquito midgut microbiota [14,15,17], it remains unclear whether it also is influenced by mosquito genotype[25]. To test for a role of mosquito genotype, we investigated whether adult females from multiple strains of *A*. *aegypti* vary in their midgut microbial load when reared under identical conditions. We reared five strains of *A*. *aegypti* [33] in parallel in the laboratory, mixing the rearing water three times between the strains during development to ensure exposure to highly similar rearing water microbiota. We analyzed the LB-cultivable bacterial composition and load of the larval rearing water at the 4^th^ larval instar and pupal stage and found both to be nearly identical between the strains (S1 Fig; L4 bacterial load, p = 0.1991; pupal bacterial load, p = 0.9928). We either maintained adult females on 3% sterile sucrose or provided a sterile blood meal, and at forty-eight hours post blood feeding we assayed total LB-cultivable midgut bacterial load (Fig 1). We used a culture-dependent method for this experiment because it allowed us to assay only live bacteria.

We found that total adult female midgut bacterial load differed significantly among the *A*. *aegypti* strains (Fig 1). We analyzed these data using a zero-inflated data analysis because multiple samples harbored zero LB-cultivable microbes. For count data (*i*.*e*. samples with at least one CFU), the effect of strain was highly significant (p = 6.19 × 10^−14^, S3 Table), suggesting that when LB cultivable bacteria are present, the number of bacteria varies significantly between strains. For presence/absence data (*i*.*e*. zero vs. at least one CFU), we detected a significant interaction between strain and feeding status (p = 0.0174, S3 Table). This suggests that the proportion of individuals lacking LB-cultivable bacteria differs between the strains in a manner that is feeding-status dependent. This is illustrated in Fig 1 and S4 Table, where the differences between strains in the proportion of “zero” values are much more pronounced after blood feeding. These observed differences in bacterial load between strains are unlikely to be due to variability in the pre-adult rearing environment, since the strains were exposed to highly similar bacterial communities during development (S1 Fig). In summary, our results indicate that female midgut bacterial load differs significantly among strains of *A*. *aegypti*, both before and after blood feeding.

### Midgut bacterial load significantly differs between Rock and Sing strains over time

**Fig 2:**
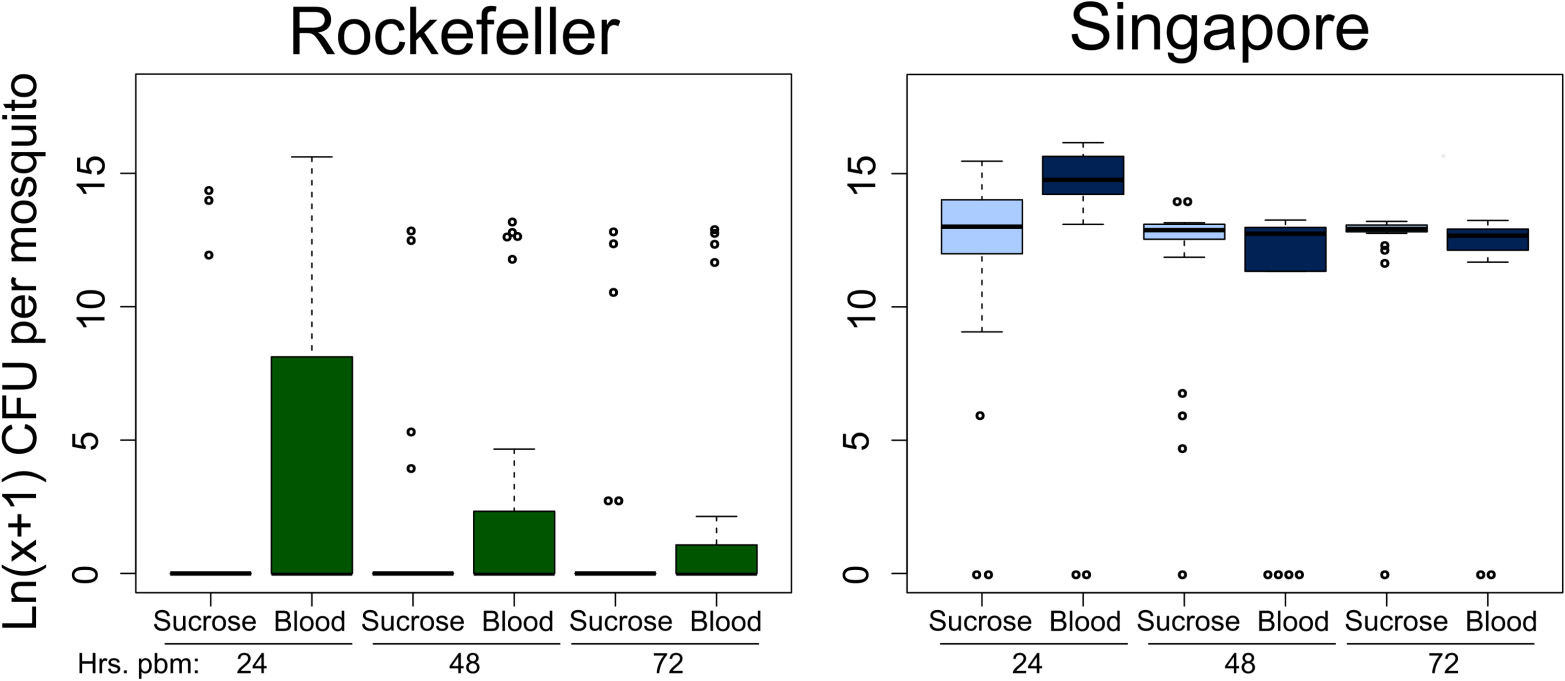
Rockefeller female midguts have lower bacterial loads than Singapore females at multiple time points post blood feeding. We reared female *A*. *aegypti* from Rockefeller and Singapore strains in parallel and mixed the breeding water between strains three times during larval/pupal development. We maintained adults on 3% sucrose until 5 days post eclosion, at which point a subset of the females were given a sterile blood meal. At 24, 48 and 72 hours post blood feeding, we externally sterilized sugar and blood fed females from each strain with 70% EtOH, dissected midguts from each strain/feeding treatment, homogenized individual midguts in 1X PBS using a sterile pestle and diluted the homogenate 1:100 and 1:10^4^ using additional 1X PBS. We spread 50ul of each dilution as well as undiluted homogenate on LB agar plates, allowed the plates to grow for 48 hours at room temperature and then counted the total CFUs on each plate. We performed a zero-inflated data analysis to test the effect of strain, feeding status and time post blood feeding, as well as all pairwise interactions and a three-way interaction between factors. We performed stepwise backwards selection of model terms using likelihood ratio tests to assess the significance of each dropped term. For count data, strain was a significant predictor of bacterial load (p = 0.0237) and we also detected a significant interaction between feeding status and time post blood feeding (p = 0.0060). For presence/absence data, we detected a significant interaction between strain and feeding status (p = 0.0390). Time post blood feeding was not a significant predictor for presence/absence data. Data were collected over three experimental replicates and total sample sizes are as follows: n_rock.sug.24_ = 23; n_rock.sug.48_ = 23; n_rock.sug.72_ = 21; n_sing.sug.24_ = 24; n_sing.sug.48_ = 24; n_sing.sug.72_ = 23; n_rock.blood.24_ = 24; n_rock.blood.48_ = 24; n_rock.blood.72_ = 23; n_sing.blood.24_ = 21; n_sing.blood.48_ = 17; n_sing.blood.72_ = 20.

In order to test whether the differences in female midgut microbial load between the two most disparate strains, Rock and Sing, persists over time, we repeated the same experiment as in the single time point bacterial load analysis shown in Fig 1 for these two strains, but analyzed the midgut microbiota at 24, 48 and 72 hours post blood feeding (Fig 2). Sugar fed controls were collected in parallel at the same time points. We again conducted a zero-inflated data analysis, testing for three factors: strain, feeding status and time post blood feeding. We tested for a three-way interaction between these factors as well as two-way interactions between each pairwise combination of factors (S5 Table). For individuals with at least one CFU, we found that bacterial load differed significantly between strains (p = 0.0237) and we also detected a significant interaction between feeding status and time post blood feeding (p = 0.0060), meaning that the effect of blood feeding varied over time (Fig 2, S5 Table). While bacterial loads increased in both strains at 24 hours, there was little effect of blood feeding on bacterial load by 48 hours. This is consistent with findings by others showing that most bacterial proliferation after a blood meal occurs in the first 24 hours post feeding [27,28]. We note that, despite an increase in bacterial load at 24 hours after blood feeding, Rock strain bacterial load levels are consistently lower than Sing strain levels and rapidly drop off after this time point, returning to near zero levels by 48 hours, similar to what was observed in our single time point bacterial load analysis (Fig 1). For presence/absence data, we detected a significant interaction between strain and feeding status (p = 0.0390), meaning that the proportion of individuals in each strain with zero CFUs depended on feeding status (Fig 2, S5 Table). A larger proportion of Rock strain sugar fed females had undetectable microbiota than Sing strain sugar fed females, while for blood fed females the difference between strains in the number of individuals with undetectable midgut bacteria is less extreme (Fig 2). Despite this, Rockefeller strain had a higher number of females with undetectable midgut microbiota than Sing strain females at all time points and for both feeding statuses (Fig 2). Taken together, these data show that Rock strain females have consistently lower bacterial loads than Sing strain females. Though they do display an increase in midgut bacterial loads after blood feeding, this increase quickly drops off over time and the LB-cultivable number of bacteria returns to near zero. This is in stark contrast to Sing strain females, who maintain very high midgut microbial loads over time.

### Culture independent method confirms lower midgut bacterial load in Rock strain females compared to Sing strain females

**Fig 3:**
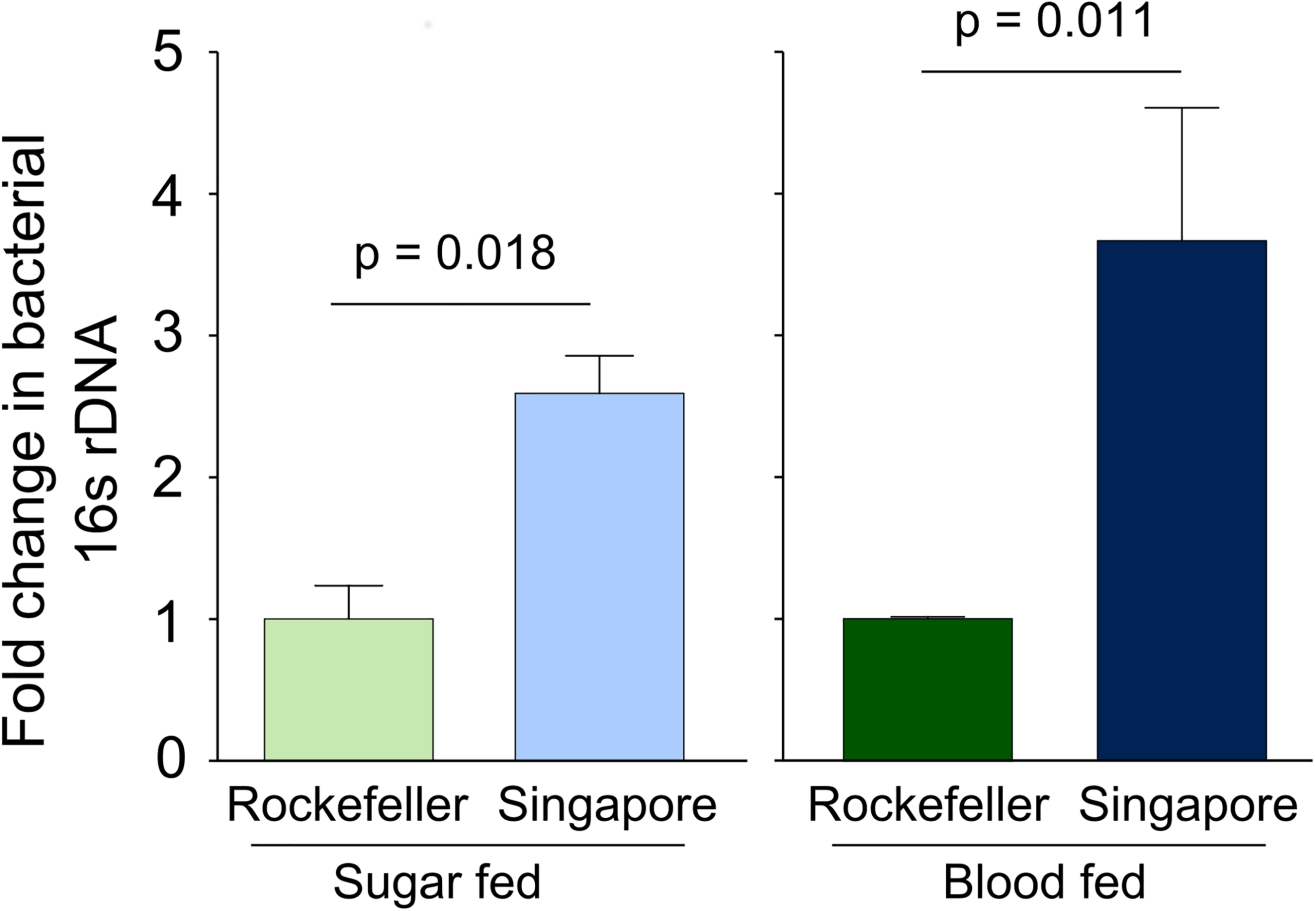
Rockefeller midguts have relatively lower bacterial 16S gene levels compared to Singapore midguts as measured by qPCR. We reared female Rockefeller and Singapore strain *A*. *aegypti* in parallel and mixed the breeding water between the strains three times during larval/pupal development. Adults were maintained on 3% sucrose upon eclosion. At 3–5 days post eclosion, females were either provided a sterile blood meal or sterile sucrose. Twenty-four hours post blood meal, we externally sterilized all females by washing with 70% EtOH and then dissected two pools of 8 midguts in sterile 1X PBS for each strain and feeding treatment (5–6 midguts were collected for 3 Singapore blood fed samples). Sugar fed individuals were collected from three independent replicate experiments and blood fed individuals were collected from two. We extracted DNA from each midgut pool and used qPCR to quantify levels of the bacterial16S rDNA gene and *A*. *aegypti* S7 reference gene. We averaged delta Ct values from pools from the same biological replicate before analysis to prevent pseudoreplication. Values in the figure were calculated using the delta delta Ct method, where Singapore is shown relative to Rockefeller within each feeding treatment. Error bars represent one standard error. Raw delta Ct values were analyzed in R by ANOVA followed by a Tukey’s test using the following model: Y_ijk_ = μ + strain_j_ + feeding status_k_ + strain_j_ * feeding status_k_. Both strain (p = 0.00064) and feeding status (p = 3.22 x 10^−6^) were highly significant and we failed to detect an interaction between strain and feeding status.

While our live bacteria enumeration assays clearly show lower bacterial loads in the midgut of Rock strain females, culture dependent methods exclude detection of many microbes. With this in mind, we also assayed total bacterial load in sucrose and blood fed Rock and Sing strain female midguts at 24 hours post blood feeding using qPCR targeting the bacterial16S rRNA gene (Fig 3). Consistent with our findings using the live bacteria enumeration method, our qPCR-based assays also showed that in both sucrose and blood fed treatments, Rock strain females had lower relative 16S DNA copies compared to Sing strain females (sucrose fed, p_strain_ = 0.018; blood fed, p_strain_ = 0.011).

Using both culture–dependent and–independent methods, we have shown significant differences in female midgut bacterial load between strains of *A*. *aegypti*. The strains used in these experiments were collected from different parts of the globe [33], and had been maintained at identical laboratory conditions for at least two years prior to the time of this experiment. The fact that we observe significantly different bacterial loads between the strains when they were reared in a controlled laboratory environment suggests that they harbor genetic variation for factors that influence midgut microbial load. This suggests that, in addition to environment [15,17], mosquito genotype also has the potential to influence the midgut microbiota, which could in turn influence vector competence [9]. Importantly, Sing strain females have been reported to be significantly less susceptible to dengue virus serotype 4 infection as measured by viral titer and infection prevalence, and to dengue virus serotype 2 as measured by infection prevalence (though not viral titer, [33]). It is therefore possible that higher bacterial load levels in the Sing strain midgut contribute to this difference in vector competence. However, further work is necessary to address the influence of mosquito genotype on the midgut microbiota in the field and any consequent implications for vector competence.

### Composition of Rock and Sing strain midgut microbiota using culture dependent and independent methods

The differences we detected in bacterial load between the mosquito strains could potentially be attributed to: (1) a universal effect on bacterial load, in which the bacterial population as a whole proliferates more in some mosquito strains than in others, (2) a species-specific effect on bacterial load, in which only certain bacterial species differentially proliferate, or (3) an effect on composition of the midgut, in which different bacterial species colonize certain mosquito genotypes. In the third scenario, differences in bacterial load would manifest if some species of bacteria inherently proliferate more readily in the mosquito midgut. To explore these different possibilities and to better understand the nature of the observed bacterial load variation, we next analyzed the midgut bacterial composition of females from Rock and Sing strains by assaying live bacterial colonies as well as high-throughput sequencing of the 16S rRNA gene.

**Fig 4:**
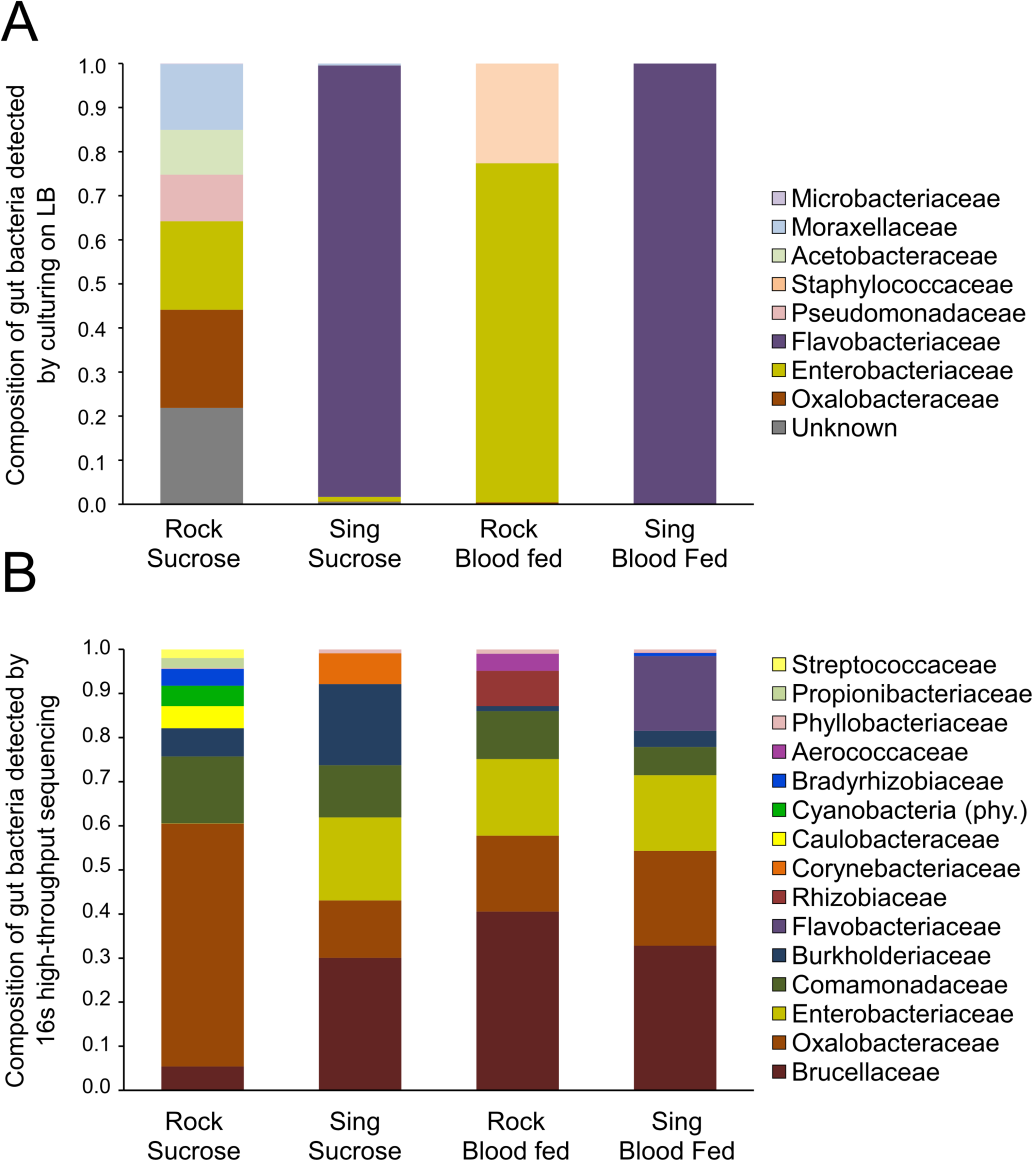
Family-level bacterial composition of Rockefeller and Singapore female midguts. **(A) Family level composition of LB-cultivable bacteria in Rock and Sing:** Using the same samples shown in the single time point bacterial load analysis presented in Fig 1, we identified distinct bacterial colony types cultured from each individual female midgut and sequenced the 16S rRNA gene from each isolate to identify colony type to family level. Bacterial family counts from individual females were combined *post hoc* within each combination of strain and feeding status to obtain an overall percentage of bacterial family within each treatment group. LB-cultivable composition data for the other three strains shown in Fig 1 can be found in S2 Fig. **(B) Family level composition using 16S rRNA high-throughput sequencing.** We used Illumina MiSeq technology to sequence the V3-V4 hypervariable regions of the 16S rRNA gene from a pooled subset of samples from those used in the single time point bacterial load analysis presented in Fig 1 to determine their family-level 16S midgut bacterial community profile. Only OTUs representing >1% of the total reads are represented here. Cyanobacteria are presented at the phylum level, as reads could not be classified accurately below this level.

We determined the composition of the midgut bacterial community of each strain using the same samples as in the single time point bacterial load analysis (Fig 1). To assess composition of the LB-cultivable (live) bacteria in Rock and Sing strain midguts, we first characterized and counted distinct bacterial colony types cultured from individual mosquito midguts using standard microbiological techniques. We then determined the bacterial species of each colony type based on 16S rRNA gene sequence analysis. Colony counts for each bacterial family cultured from Rock and Sing strain females were combined from all individual mosquito samples within each treatment to determine an overall proportion of each bacterial family in each treatment group (Fig 4A) (Composition data for the remaining strains presented in the single time point bacterial load analysis (Fig 1) can be found in S2 Fig).

The data reveal that the vast majority of LB-cultivable bacteria in Sing strain female midguts from both sucrose and blood fed groups belonged to the Flavobacteriaceae family, and all bacteria from this family were identified as *Elizabethkingia meningoseptica*. *Elizabethkingia* are commonly found in mosquito midguts, and have been detected both in laboratory and field mosquitoes [29,58–60]. Interestingly, *E*. *meningoseptica* was absent from Rock strain samples, suggesting that differential prevalence of this bacteria is primarily responsible for the dramatic difference we observed in LB-cultivable midgut microbial loads (Fig 1). Approximately 20% of Rock individuals in the multiple time point bacterial load experiment (Fig 2) harbored *E*. *meningoseptica*. We assessed bacterial loads of this species over time in both Rock and Sing strain females and found that bacterial loads of this species decreased over time in Rock strain females but remained quite high in Sing strain females (S3 Fig). At 24 hours post blood feeding, the number of *E*. *meningoseptica* in midguts of both strains did not significantly differ (S3 Fig, p = 0.5127). By 48 hours, we detected a trend toward a difference between the strains (S3 Fig, p = 0.06856) which became more pronounced and highly significant by 72 hours post blood feeding (S3 Fig, p = 0.0001). This suggests that even when *E*. *meningoseptica* is present, Rock strain midguts are less amenable to its maintenance over time. Together, these data suggest that genetic differences between these strains may influence colonization of the midgut by at least one species of bacteria, *E*. *meningoseptica*, as well as persistence of these bacteria over time.

To assess the composition of the midgut microbiome of Rock and Sing strain females at a higher resolution, we employed high-throughput sequencing of the V3-V4 hypervariable regions of the bacterial 16S rRNA gene on the same samples used in the single time point bacterial load experiment (Fig 4B). As expected, this culture independent method revealed much higher diversity in the midgut microbiota of all strain/treatment combinations compared to our culture-dependent method, with sequencing detecting 15 bacterial families and culturing detecting only 8 (Fig 4). Consistent with the culture-dependent data, *Flavobacteriaceae* was only detected in Sing strain females, but at very low levels (<1% total reads) in the sucrose-fed mosquitoes while representing 17% of the total reads in blood fed females (Fig 4B, S1 File).

The sequencing-based assays also revealed that the midgut bacterial composition was much more similar between the strains than was originally suggested by culture-dependent assays. Weighted UniFrac analysis revealed little differentiation between most samples, with values ranging from 0.2–0.3 (Table 1). The only exceptions were comparisons between Rock sucrose and both blood fed treatments, where UniFrac values were higher (Table 1, Rock sucrose vs. Rock blood = 0.406, Rock sucrose vs. Sing blood = 0.448), suggesting that sucrose fed Rock females have a midgut microbiome that is comparatively more dissimilar from either blood fed sample than any other pairwise comparison. The Rock strain sucrose fed females showed a higher proportion of *Oxalobacteraceae* compared to other treatments, coupled with a near absence of *Entobacteriaceae* (Fig 4B). Nonmetric Multidimensional Scaling (NMDS) ordination performed using Bray-Curtis dissimilarity values validated these results; Rock sucrose fed was most dissimilar from Sing blood fed followed by Rock blood fed, and most similar to Sing sucrose fed (S4 Fig). This pattern is likely attributable to the general absence of *Entobacteriaceae* in Rock strain sucrose fed females as well as the high abundance of *Flavobacteriaceae* in Sing strain blood fed females (S4 Fig).

**Table 1:** Weighted UniFrac distances between Rock and Sing microbiome samples.

	Rock Sucrose	Sing Sucrose	Rock Blood	Sing Blood
Rock Sucrose	0	0.281	0.406	0.448
Sing Sucrose		0	0.209	0.266
Rock Blood			0	0.207
Sing Blood				0

Overall, the culture-independent data suggest that the composition of the midgut microbiome is quite similar between strains and especially within feeding treatments. This is particularly true for Rock and Sing strain blood fed samples, which shared the highest degree of similarity of all the samples (UniFrac = 0.207). High similarity in composition suggests that differences in midgut bacterial load obtained using culture independent qPCR-based studies (Fig 3) are less likely to be due to differences in species-specific bacterial proliferation, especially among blood fed females, and more likely to be due to general proliferation of the total midgut microbiome. We cannot rule out the possibility that cumulative minor differences in composition could contribute towards bacterial load differences between the strains (*e*.*g*. that of *Flavobacteriaceae*), but given the overall similarity in composition between the strains, we consider it more likely that Rock strain female midguts may be generally less amenable to bacterial proliferation or survival in a manner that broadly affects bacterial load of many bacterial species.

### Rock and Sing females differ in transcript abundance of many metabolism-related genes

**Fig 5:**
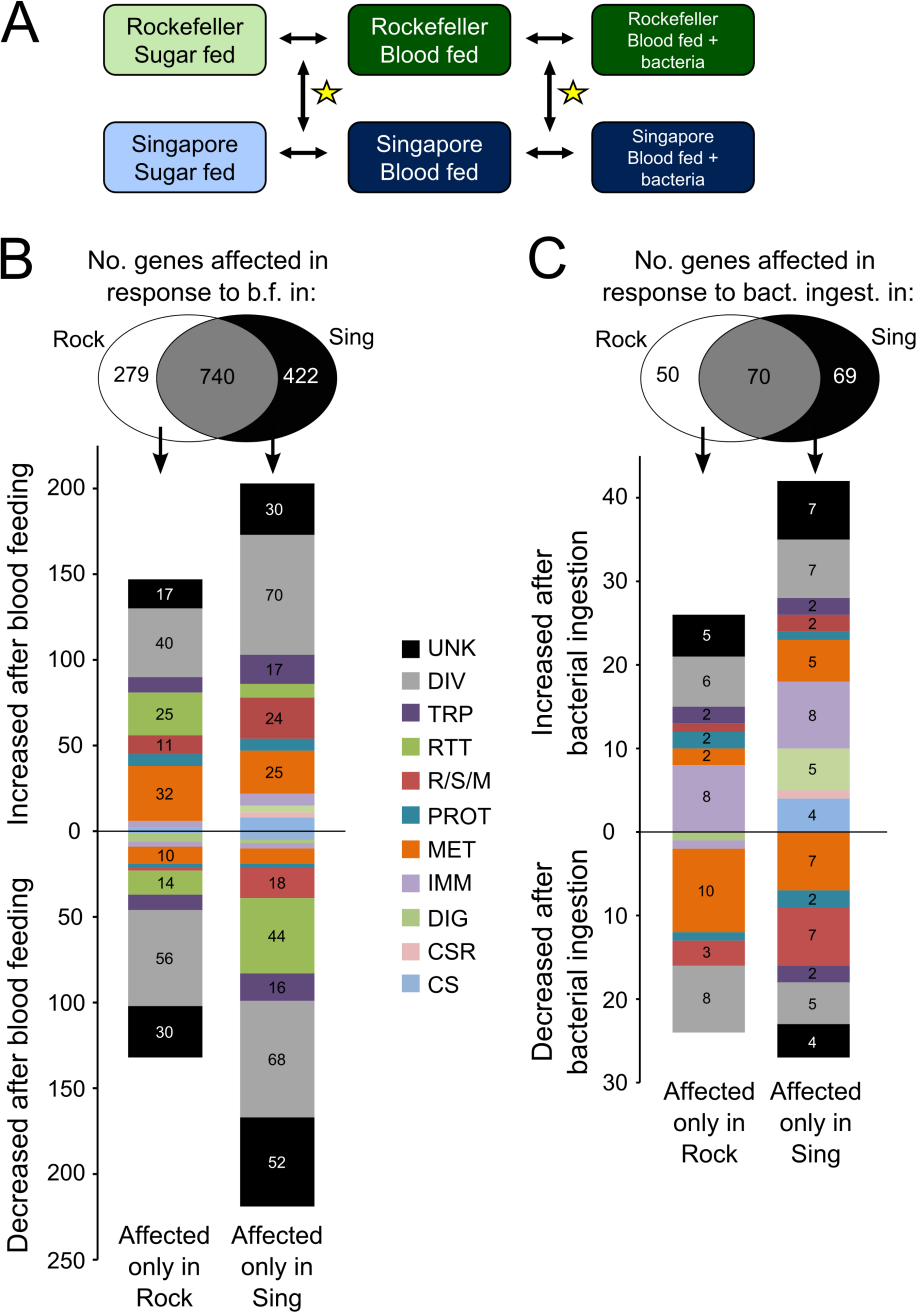
Genes showing significant changes in transcript abundance in response to blood feeding or bacterial re-introduction in either Rockefeller or Singapore female midguts. **(A) Experimental design to measure gene expression differences between Rockefeller and Singapore female midguts.** Rockefeller and Singapore females were reared in parallel and provided with 3% sucrose + antibiotics (pen/strep + gent) upon eclosion. At 5–7 days post eclosion, females were maintained on sucrose, given a sterile blood meal, or given a blood meal spiked with a mixed culture of seven common mosquito midgut bacteria (S2 Table). Midguts were dissected from each strain/feeding treatment 12 hours post blood feeding and genome wide gene expression was measured for each strain/feeding treatment relative to a pooled reference sample using custom Agilent gene expression microarrays. Comparisons of interest in this experiment (indicated by black arrows) are as follows: Rock sucrose vs. Rock blood fed, Sing sucrose vs. Sing blood fed, Rock blood fed vs. Rock bacteria fed, Sing blood fed vs. Sing bacteria fed. We were also interested in how response to treatment differed between the strains (yellow stars), *i*.*e*. transcript changes observed in one strain that are smaller or absent in the other strain. **(B) Strain-specific changes in transcript abundance in response to blood feeding.** Change in transcript abundance in response to blood feeding (*i*.*e*. sucrose fed vs. blood fed) was measured for both Rockefeller and Singapore females. Genes that responded to treatment in either or both strain(s) are shown in the Venn diagram. Genes that increased or decreased in transcript abundance in only Rockefeller or only Singapore (*i*.*e*. strain-specific responses) are shown in the bar graphs along with functional categorization data based on gene ontology. **(C) Strain-specific changes in transcript abundance in response to bacterial re-introduction.** Change in transcript abundance in response to bacterial re-introduction (*i*.*e*. blood fed vs. blood + bacteria fed) was measured for both Rockefeller and Singapore females. Genes that responded to treatment in either or both strain(s) are shown in the Venn diagram. Genes that increased or decreased in transcript abundance in only Rockefeller or only Singapore (*i*.*e*. strain-specific responses) are shown in the bar graphs along with functional categorization data based on gene ontology. UNK = unknown function, DIV = diverse functions, TRP = transport, RTT = replication, transcription, and translation, R/S/M = redox, stress and mitochondrion, PROT = proteolysis, MET = metabolism, IMM = immunity, DIG = blood and sugar digestion, CSR = chemosensory reception, CS = cytoskeletal and structural.

In order to begin to understand why Rock and Sing strain females differ so substantially in midgut bacterial load, we performed a microarray-based whole-genome transcriptome analysis to study changes in mRNA transcript abundance in midguts of the strains after multiple feeding treatments (Fig 5). We reared both strains in parallel and upon eclosion treated adult females with antibiotics to significantly reduce their native midgut microbiota [61]. We reasoned that without this step, differences in transcript abundance could arise simply as a response to standing variation in midgut microbial load. By treating with antibiotics, we aimed to maximize our detection of gene expression differences that are causal of, rather than simply a response to, midgut bacterial load variation. We prepared samples from midguts of females of each strain that had been either maintained on 3% sucrose, given a sterile blood meal 12 hours earlier, or given a blood meal containing a cocktail of commonly isolated midgut bacteria12 hours earlier (S2 Table). We then hybridized all samples against a common reference sample, and made pairwise comparisons between treatments (Fig 5A). To assess differences in the way that each strain responded to treatment, we queried for genes that changed significantly in response to treatment in only one strain (Fig 5B).

In response to blood feeding, transcript abundance of 279 genes changed significantly in Rock strain female midguts but not in Sing strain females (Rock-specific changes), while 422 genes changed significantly in Sing strain females but not in Rock strain females (Sing-specific changes). Rock strain-specific transcript abundance changes included many putative metabolism-related genes that were up-regulated in response to blood feeding (Fig 5B). Biological process Gene Ontology (GO) analysis on this same gene set also showed many GO terms related to metabolism were significantly enriched (Table 2). In particular, the GO term “carboxylic acid catabolic process” (GO0046395) was the most significantly enriched. Carboxylic acids are those molecules that contain a carboxyl group, including acetic acid, fatty acids and amino acids. We performed a KEGG pathway analysis on this Rock strain-specific gene set and found that the KEGG pathway “valine, leucine and isoleucine degradation” (aag00280) was significantly enriched (Table 3). Upon further inspection, we found that 25% of the genes in this pathway (7 of 28) showed significantly increased transcript abundance in Rock but not in Sing strain female midguts. Additionally, multiple KEGG pathways involved in fatty acid metabolism were also enriched (Table 3).

**Table 2:** Biological process gene ontology terms enriched for genes showing transcript abundance changes in either Rockefeller or Singapore in response to blood feeding or bacterial reintroduction.

GO ID	GO name	Expected Count	Count	Size	p-value	BH adj. p-value
**GO terms enriched for genes responding to blood feeding in Rockefeller but not in Singapore**						
GO:0046395	carboxylic acid catabolic process	0.562	6	26	1.47E-05	0.0034
GO:0044282	small molecule catabolic process	0.648	6	30	3.53E-05	0.0042
GO:0006082	organic acid metabolic process	4.646	15	215	5.49E-05	0.0042
GO:0042180	cellular ketone metabolic process	4.840	15	224	8.77E-05	0.0058
GO:0006631	fatty acid metabolic process	0.821	6	38	1.43E-04	0.0062
GO:0055114	oxidation-reduction process	15.148	30	701	1.62E-04	0.0062
GO:0034440	lipid oxidation	0.130	3	6	1.88E-04	0.0062
GO:0006081	cellular aldehyde metabolic process	0.151	3	7	3.24E-04	0.0089
GO:0032787	monocarboxylic acid metabolic process	0.951	6	44	3.28E-04	0.0089
GO:0006777	Mo-molybdopterin cofactor biosynthetic process	0.216	3	10	1.06E-03	0.0223
GO:0051189	prosthetic group metabolic process	0.216	3	10	1.06E-03	0.0223
GO:0006732	coenzyme metabolic process	2.161	8	100	1.36E-03	0.0273
GO:0006725	cellular aromatic compound metabolic process	1.253	6	58	1.47E-03	0.0283
GO:0042558	pteridine-containing compound metabolic process	0.562	4	26	2.16E-03	0.0384
GO:0051186	cofactor metabolic process	2.355	8	109	2.35E-03	0.0403
**GO terms enriched for genes responding to blood feeding in Singapore but not in Rockefeller**						
GO:0006412	translation	10.419	27	303	4.15E-06	0.0020
GO:0055114	oxidation-reduction process	24.104	46	701	8.78E-06	0.0021
GO:0071843	cellular component biogenesis at cellular level	2.269	10	66	7.33E-05	0.0088
GO:0042254	ribosome biogenesis	1.513	8	44	1.05E-04	0.0101
GO:0001522	pseudouridine synthesis	0.413	4	12	5.42E-04	0.0434
**GO terms enriched for genes responding to bacterial reintroduction in Rockefeller but not in Singapore**						
GO:0006082	organic acid metabolic process	1.085	7	215	7.64E-05	0.0026
GO:0042180	cellular ketone metabolic process	1.131	7	224	9.90E-05	0.0026
GO:0046394	carboxylic acid biosynthetic process	0.363	4	72	4.32E-04	0.0076
GO:0044283	small molecule biosynthetic process	0.399	4	79	6.16E-04	0.0092
GO:0044281	small molecule metabolic process	3.372	9	668	4.57E-03	0.0408
GO:0009089	lysine biosynthetic process via diaminopimelate	0.005	1	1	5.05E-03	0.0408
**GO terms enriched for genes responding to bacterial reintroduction in Singapore but not in Rockefeller**						
GO:0070981	L-asparagine biosynthetic process	0.012	2	2	3.69E-05	0.0034
GO:0006528	asparagine metabolic process	0.018	2	3	1.10E-04	0.0051
GO:0006790	sulfur compound metabolic process	0.234	3	38	1.56E-03	0.0287
GO:0006082	organic acid metabolic process	1.323	6	215	1.81E-03	0.0287
GO:0005975	carbohydrate metabolic process	1.833	7	298	1.98E-03	0.0287
GO:0008652	cellular amino acid biosynthetic process	0.258	3	42	2.09E-03	0.0287
GO:0042180	cellular ketone metabolic process	1.378	6	224	2.23E-03	0.0287
GO:0009066	aspartate family amino acid metabolic process	0.074	2	12	2.34E-03	0.0287

**Table 3:** KEGG pathways enriched among genes showing changes in transcript abundance in either Rock or Sing in response to blood feeding or bacterial reintroduction.

KEGG ID	KEGG Pathway Name	p-value	BH adjusted p-value
**Genes affected by blood feeding in Rockefeller but not Singapore females.**			
aag00280	Valine, leucine and isoleucine degradation	0.00004	0.00174
aag00071	Fatty acid metabolism	0.00017	0.00390
aag00062	Fatty acid elongation in mitochondria	0.00151	0.02235
aag01040	Biosynthesis of unsaturated fatty acids	0.00267	0.02961
aag00650	Butanoate metabolism	0.00757	0.06610
aag03050	Proteasome	0.01425	0.10208
aag00310	Lysine degradation	0.01574	0.09696
**Genes affected by blood feeding in Singapore but not Rockefeller females.**			
aag03010	Ribosome	0.00003	0.00129
aag03450	Non-homologous end-joining	0.02322	0.45072
aag00020	Citrate cycle (TCA cycle)	0.02706	0.37270
**Genes affected by bacterial reintroduction in Rockefeller but not Singapore females.**			
NONE			
**Genes affected by bacterial reintroduction in Singapore but not Rockefeller females.**			
aag00250	Alanine, aspartate and glutamate metabolism	0.02561	0.40485

Previous work has shown that upregulation of genes involved in amino acid catabolism and fatty acid metabolism occurs after a blood meal in *A*. *aegypti* [62]. This occurs in order to utilize proteins and lipids present in the blood, which are necessary for egg production [63]. Differences in expression of these genes between the Rock and Sing strains suggest that they may differ in their absorption or utilization of lipids and proteins from the blood meal. In particular, expression of genes controlling branched-chain amino acid (valine, leucine and isoleucine) degradation differs between the strains (Table 3). The branched-chain amino acids (BCAAs) are essential amino acids for mosquitoes, meaning they cannot synthesize them and must therefore obtain them from their diet [64,65]. In addition to serving as building blocks for protein production, BCAAs are degraded in the cell in order to produce acetyl-CoA and succinyl-CoA for use in cellular respiration [66]. Furthermore, BCAAs and their degradation products–branched chain α-keto acids (BCKAs)–are signaling molecules which activate the major nutrient signaling pathway Target of rapamycin (TOR) [67,68]. TOR signaling regulates many basic cellular processes in response to nutrient availability, including cellular growth, autophagy, and mRNA translation [69,70]. In mosquitoes, BCAA activation of TOR promotes expression of the yolk protein precursor gene *vitellogenin* [71,72], and in *Drosophila*, TOR has been shown to regulate expression of multiple antimicrobial peptide genes, which are the main effector molecules of the humoral immune response [73].

In examining Sing strain-specific changes in gene expression, we found many genes belonging to the replication, transcription and translation functional group to be down-regulated in response to blood feeding (Fig 5B). GO analysis revealed that the term “translation” (GO0006412) was significantly enriched in this gene set (Table 2), and KEGG pathway analysis showed gene transcripts relating to the “ribosome” (aag03010) pathway to be enriched (Table 3). A decrease in expression of translation-related genes in response to blood feeding has been reported in *A*. *aegypti* previously, and it is thought that this is part of a generalized stress response of the midgut [62,74]. The fact that this phenomenon is so much more pronounced in Sing strain compared to Rock strain females suggests that Sing females may perhaps respond more effectively to cellular stress after a blood meal.

In response to bacterial ingestion, transcript abundance of 50 genes was altered significantly in Rock but not in Sing strain females while transcripts of 69 genes were altered significantly in Sing but not in Rock strain females (Fig 5B). Rock strain-specific transcript abundance changes again reflected the potential influence of metabolic processes, including carboxylic acid metabolism (Table 2). Sing strain-specific transcript abundance changes also implicated metabolic processes, specifically asparagine metabolism (Tables 2 and 3). In addition to genes involved in metabolism, we also found that many immune-related genes showed differential mRNA abundance between the strains (Fig 5B). In Rock strain females, eight immunity genes responded to bacterial ingestion in a Rock strain-specific manner. These included five serine proteases and a serine protease inhibitor (S1 File). Serine proteases play important roles in regulating multiple components of immune signaling, including the melanization cascade and the Toll pathway, both of which produce anti-microbial effector molecules [75,76]. The Toll pathway is also an important component of the mosquito immune defense against dengue virus [9]. Serine proteases also regulate digestion, however [77], and the specific function of the Rock strain differentially regulated enzymes is not clear. Differential immune system signaling could affect bacterial proliferation through production of anti-microbial peptides or other anti-microbial molecules such as the toxic quinones produced during melanin synthesis. Digestion regulation could also potentially influence midgut bacterial load by affecting nutrient availability in the midgut. In Sing females, eight immunity-related genes responded to bacterial ingestion in a Sing strain-specific manner. These included the immune gene *cactus* as well as two genes coding for fibrinogen and fibronectin-domain containing proteins (S1 File). *Cactus* is a negative regulator of the Toll immune pathway [9,78,79], which plays an important role in mosquito immune defense against dengue virus [9] and also acts to inhibit the complement-like pathway, which protects against both parasites and bacteria in mosquitoes [80,81]. Fibrinogen-domain containing proteins serve as pattern recognition receptors and are important for anti-bacterial and anti-parasitic immunity in mosquitoes [82].

### Silencing the valine-leucine-isoleucine degradation pathway eliminates bacterial load differences between strains but does not alter dengue virus titer in the midgut

**Fig 6:**
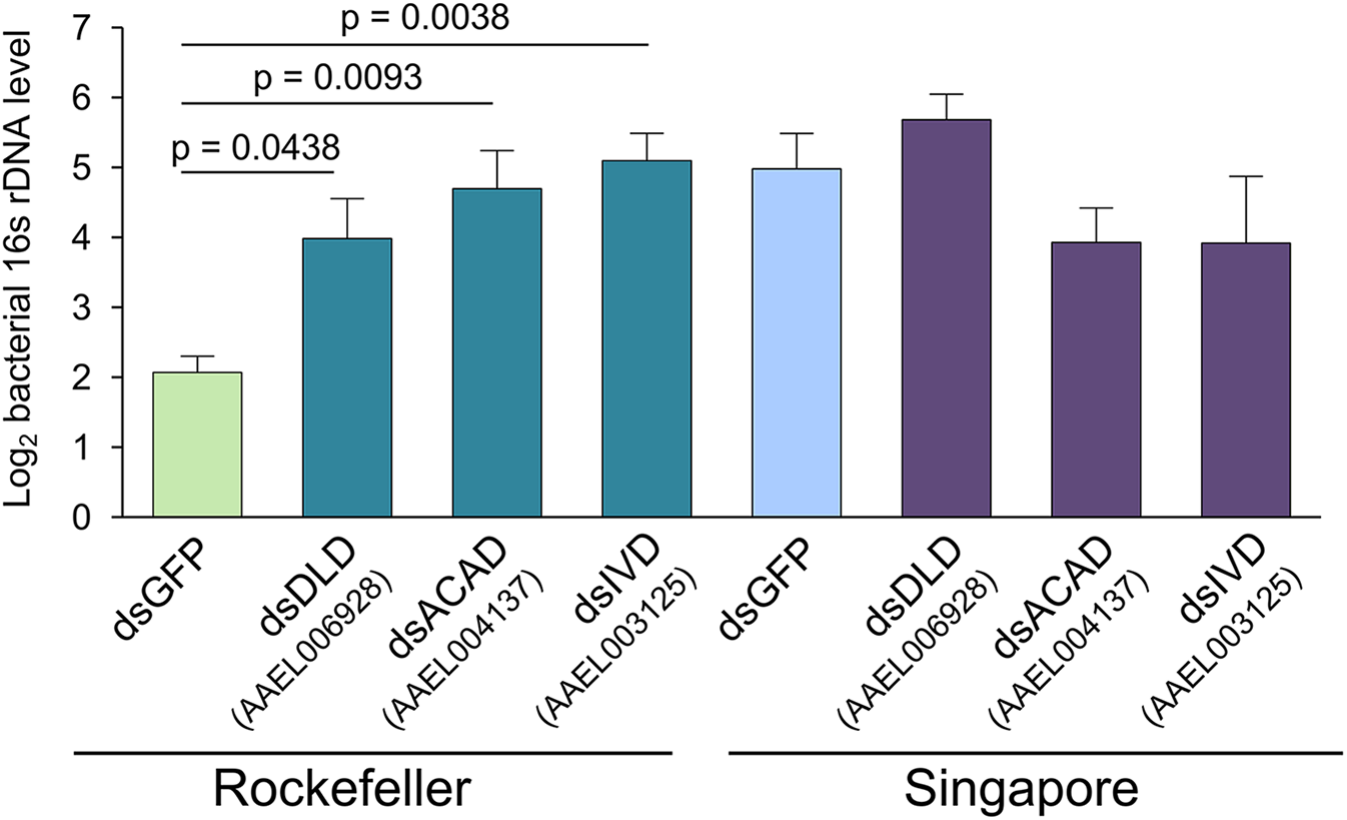
RNAi silencing of genes involved in Val-Leu-Ile degradation causes Rockefeller-specific increases in relative bacterial 16S rDNA. We reared Rockefeller and Singapore mosquitoes in parallel and injected them at 3–5 days post-eclosion with 200ng of dsRNA targeting one of the three experimental genes or eGFP as a control. At two days post injection, we dissected two pools of eight midguts for each strain/treatment combination. This entire experiment was repeated three independent times. We extracted DNA from each midgut pool and performed qPCR to quantify levels of the bacterial16S rDNA gene and *A*. *aegypti* S7 reference gene. We averaged delta Ct values from pools from the same biological replicate before analysis to prevent pseudoreplication, and Y-axis values are average inverse delta CT values, i.e. -1*(CT_16S_ –CT_S7_) for each treatment. Because CT values are Log_2_, a difference of 1 on the y-axis corresponds to a 2-fold change in 16S DNA abundance. Error bars represent one standard error. Raw delta CT values were analyzed in R by ANOVA followed by a Dunnett’s test using the following model: Y_ijk_ = μ + strain_j_ + treatment_k_ + strain_j_ * treatment_k_. We detected a significant interaction between strain and treatment (p = 0.0044), and using a Dunnett’s test found that silencing all three genes caused a significant increase in 16S rRNA gene levels relative to Rock GFP-injected controls. No significant effects of silencing were detected in Sing females. DLD = dihydrolipoamide dehydrogenase, ACAD = acyl-CoA dehydrogenase, IVD = isovaleryl-CoA dehydrogenase.

Our genome-wide transcriptome analyses strongly suggested that metabolic signaling differs between Rock and Sing strain females. We therefore decided to investigate whether relevant metabolic pathways play a causal role in controlling midgut microbial load. We chose to focus on the BCAA degradation pathway, because it was indicated as being enriched by both GO analysis and KEGG pathway analysis. Additionally, multiple genes in this pathway are differentially expressed between the strains, suggesting a robust and concerted difference in pathway activity. In order to functionally test the role of the BCAA degradation pathway on midgut bacterial load, we silenced multiple genes from this pathway in both strains and measured relative levels of the bacterial 16S rRNA gene using qPCR. Silencing efficiency for the three pathway genes (dihydrolipoamide dehydrogenase, an acyl-CoA dehydrogenase, and isovaleryl-CoA dehydrogenase) was successful in both strains (S5 Fig), and resulted in an increase in midgut bacterial load in Rock strain females thereby eliminating the difference in bacterial load between the two strains (Fig 6).

These data suggest that strain-specific differences in regulation of the BCAA degradation pathway may in part explain the differences in bacterial load, though the mechanism by which this occurs remains unclear. One potential hypothesis is that BCAA degradation activity in the mosquito gut influences BCAA levels, which have a consequent effect on bacterial load. BCAAs can be used as an energy source for bacteria [83], and Rock strain midguts may have lower availability of BCAAs leading to slower relative bacterial proliferation. Differences in BCAA levels between the strains could also affect midgut bacterial load through BCAA-mediated cellular signaling. As mentioned above, BCAAs and their primary degradation products BCKAs activate the TOR pathway. TOR pathway activation has been shown to cause reduced expression of the antimicrobial peptide (AMP) genes *Diptericin* and *Metchnikowen* in *D*. *melanogaster* [73], and lower AMP gene expression has been shown to correlate with higher midgut bacterial loads in *Anopheles gambiae* mosquitoes [22,32]. Differences in transcript levels of BCAA degradation genes, either between strains or as a result of gene silencing, could therefore potentially influence gut microbial load via altered AMP production.

To begin to explore these hypotheses, we tested whether BCAA levels in midgut tissue differ between Rockefeller and Singapore strain females. We dissected midguts from Rock and Sing strain females 5–7 days post eclosion and subjected the tissue to amino acid analysis to determine the levels of valine, leucine, and isoleucine (S6 Fig). We found no difference in the molar fraction of any of these amino acids between the Rock and Sing strain midguts (S6 Fig), suggesting the strains do not differ in availability of BCAAs in the midgut. We also tested the role of BCAA availability in the mosquito midgut by feeding BCAAs to Rock and Sing females in a sugar meal. We predicted that BCAA ingestion would recapitulate the results in Fig 6, causing bacterial proliferation in the Rock midgut. We found no effect of BCAA ingestion on midgut bacterial load in either strain (S7 Fig). We note that our amino acid analysis does not distinguish between amino acids in gut bacteria versus those derived from mosquito tissue specifically, which could potentially obscure or overwhelm a difference between the strains. It is also possible that the microbiota may only be influenced by BCAA levels above a specific threshold, or that a specific amino acid ratio is required for bacterial proliferation which we failed to replicate with this experiment. Furthermore, sugar meal-mediated ingestion of BCAAs may not have been spatially distributed in the gut in a manner required for influencing the microbiota. Elucidating the mechanism by which BCAA degradation signaling influences gut bacterial load will require further investigation.

In addition to investigating the potential mechanisms by which BCAA signaling may influence gut bacterial load, we also tested whether this pathway has the potential to influence vector competence to dengue virus. We injected adult Rockefeller strain females with dsRNA targeting one of the BCAA degradation pathway genes described above (AAEL006928, AAEL004137, AAEL003125) or eGFP as a control. Two days post-injection, we provided females with a blood meal containing infectious dengue virus and we dissected midguts from individual blood fed females seven days after dengue infection. We then assayed dengue viral titers in each sample by plaque assay. We found that mean viral titer did not significantly differ between females of each treatment, and overall prevalence of infection was also similar (S8 Fig). These results suggest that the bacterial proliferation caused by silencing genes in the BCAA degradation pathway (Fig 6) is not sufficient to affect vector competence to dengue virus in the laboratory. While higher bacterial load in the midgut has been associated with lower dengue viral titer [9], additional studies have indicated that not all midgut bacteria have the same effect on vector competence. Rather, some species of bacteria caused reduced viral titers [10,11], while some have no effect [10] and some increase viral titers [13]. It is possible that the bacteria proliferating in the midgut of Rockefeller females in this experiment are not protective against dengue virus and that the influence of BCAA degradation pathway signaling on vector competence varies depending on the bacterial composition of the midgut microbiota.

### Conclusion

In this work, we investigated molecular processes that influence variation in female adult midgut bacterial load among *A*. *aegypti* strains. Using culture–dependent and–independent methods, we found significant variation in gut bacterial load between mosquito strains. Transcriptomic analysis revealed that two of these strains, Rockefeller and Singapore, also differed in transcript abundance of many genes related to branched chain amino acid metabolism. Silencing three genes in the branched chain amino acid degradation pathway resulted in a total elimination of the midgut bacterial load difference between the strains, suggesting that BCAA degradation may in part act to influence variation in midgut bacterial load. Additional experiments suggest that availability of BCAAs in the midgut does not differ between the strains nor does increased BCAA availability influence bacterial proliferation. Silencing BCAA degradation pathway genes did not influence susceptibility to dengue virus, and the implications for BCAA signaling on vector competence for dengue remain unclear. More broadly, these results suggest that genetic or physiological variation in amino acid metabolism may have important implications for the midgut microbiota of disease vector mosquitoes.

## Supporting information

S1 Fig Family-level composition and CFU/ml of LB-cultivable bacteria in larval rearing water sampled from each strain in the single time point bacterial load analysis presented in Fig 1. We sampled an aliquot of larval water from each strain at the 4^th^ instar and pupal stages of development. We also sampled an aliquot from the rearing water after it had been pooled across all strains at each stage. Pooled water was reallocated to rearing trays or pupal cups for each strain to increase similarity in bacterial environment between strains. Each sample was serially diluted and 50μL of each dilution was spread on LB agar and grown at room temperature for 48 hours. Colony types were then characterized by form, margin, elevation, color and translucency and each type was quantified for each sample. To identify each colony type, the 16S rDNA gene was sequenced and compared to existing 16S sequences via the Ribosomal Database Project. Each strain was sampled over two replicate experiments with the exception of Rockefeller and Waco pupal stage, for which samples were only successfully analyzed from a single replicate. **(A) Composition of LB-cultivable bacteria detected in larval/pupal rearing water.** 4^th^ instar larval breeding water contained eight bacterial families, all of which were detected in all strains, with the exception of the Bangkok strain, for which six of the eight bacterial families were detected. Pupal rearing water had five of seven bacterial families detected in every strain. **(B) Total number of LB-cultivable bacteria in rearing water** At the L4 stage, average total CFU/ml rearing water ranged from 3.6 × 10^7^ to 2.2 × 10^8^ and total CFU/ml did not differ significantly between rearing water of the strains (p_strain_ = 0.1991). At the pupal stage, average CFU/ml rearing water ranged from 4.09×10^7^ to 5.66 × 10^7^, and again CFU/ml did not differ significantly between the rearing water of the strains (S1 Fig, p_strain_ = 0.9928). Bkk: Bangkok, Orl: Orlando, Rock: Rockefeller, Sing: Singapore.
(PDF)

S2 Fig Family level composition of LB-cultivable bacteria in Bangkok, Orlando and Waco strains. These data were taken from the same samples used in the single time point bacterial load analysis presented in Fig 1. We identified each colony type using 16S rRNA gene sequencing and combined counts at the family level *post hoc* within each treatment group to obtain an overall percentage.
(PDF)

S3 Fig Gut bacterial load of *Elizabethkingia meningoseptica* in Rock and Sing females over time. We quantified *E*. *meningoseptica* colonies from females sampled in the multiple time point bacterial load analysis shown in Fig 2 and assessed the effect of strain and feeding status at each time point using a general linear model. At 24 hours post blood meal (pbm), we detected a significant effect of feeding (p = 4.87 x 10^−5^) but failed to detect a significant effect of strain (p = 0.513). At 48 hours pbm, neither feeding status (p = 0.967) nor strain (p = 0.069) were significant. At 72 hours pbm, strain was highly significant (p = 0.0001) while feeding status was not (p = 0.232).
(PDF)

S4 Fig Heat map showing abundance of each OTU in each sample and grouping of samples by relatedness as determined by Bray-Curtis Distances. Each row represents abundance of reads from each OTU and is labeled by family. We chose to present the data at family level because many of the OTUs could not be assigned to a genus with high confidence.
(TIF)

S5 Fig Silencing efficiency in dsRNA injected mosquitoes relative to dsGFP controls. We injected mosquitoes with 200ng dsRNA targeting one of the candidate genes or eGFP as a control and dissected pools of 8 midguts 2 days post injection. We then extracted RNA from each pool and performed qPCR to quantify the levels of each gene as well as a reference gene, S7. We assessed differences between treatment and control samples by Student’s t-test, and we used the delta delta CT method to calculate relative expression levels, where silenced samples are shown relative to the eGFP control within each strain (*i*.*e*. Rockefeller and Singapore eGFP are both standardized to 1).
(PDF)

S6 Fig Branched chain amino acid concentrations in Rockefeller and Singapore midgut tissue. We dissected midgut tissue in pools of 25 from Rockefeller and Singapore female mosquitoes 5–7 days post eclosion and analyzed amino acid composition via ion-exchange chromatography and subsequent ninhydrin reaction detection. Percent molar fraction refers to the percent of total amino acid (nmoles) that was identified to be valine, leucine, or isoleucine in each sample. The experiment was repeated three independent times, and error bars represent one standard error.
(PDF)

S7 Fig Effect of branched chain amino acid ingestion on midgut bacterial load in Rock and Sing female *A*. *aegypti*. We reared Rockefeller and Singapore mosquitoes in parallel, and at 3–5 days post-eclosion, females from each strain were given a meal of either: 3% sucrose, 3% sucrose + 1X AA, or 3% sucrose + 10X AA, where 1X AA = 0.00034g Ile + 0.00069g Val + 0.00069g Leu per 100ml 3% sucrose. After two days, we dissected one pool of eight midguts for each strain/treatment combination. This entire experiment was repeated four independent times. We extracted DNA from each midgut pool and performed qPCR to quantify levels of the bacterial16S rDNA gene and *A*. *aegypti* S7 reference gene. Y-axis values are average inverse delta CT values, i.e. -1*(CT_16S_ –CT_S7_) for each treatment. Because CT values are Log_2_, a difference of 1 on the y-axis corresponds to a 2-fold change in 16S DNA abundance. We performed an ANOVA to test the effect of strain, feeding treatment, and an interaction between these factors. Strain was highly significant (p = 7.78 x 10^−8^), but we did not detect an effect of feeding treatment (p = 0.440) nor a two-way interaction (p = 0.237).
(PDF)

S8 Fig No effect of branched chain amino acid degradation gene silencing on dengue viral titers in Rockefeller strain female midguts. Rockefeller strain females were injected with dsRNA targeting three genes in the branched chain amino acid degradation pathway (AAEL006928, AAEL004137, AAEL003125) or eGFP as a control. Forty-eight hours after dsRNA injection, females were orally infected with dengue virus via infectious blood meal. Seven days after blood feeding, midguts were dissected from females from each treatment, and viral titer was assayed for each individual by plaque assay. Analysis by one-way ANOVA revealed no significant effect of treatment relative to GFP controls. The entire experiment was replicated twice and sample sizes for each treatment are as follows: n_dsGFP_ = 47, n_dsAAEL006928_ = 48, n_dsAAEL004137_ = 47, n_dsAAEL003125_ = 48. DLD = dihydrolipoamide dehydrogenase, ACAD = acyl-CoA dehydrogenase, IVD = isovaleryl-CoA dehydrogenase.
(PDF)

S1 File Raw data for all figures. (XLSX)

S1 Table Primers used for qPCR and dsRNA synthesis. (DOCX)

S2 Table Bacteria used in blood meal for genome-wide gene expression experiment. (DOCX)

S3 Table Zero-inflated data analysis for single time point bacterial load analysis assays presented in Fig 1 to assess the effect of strain and feeding status on midgut microbial load. (DOCX)

S4 Table Prevalence of LB-cultivable midgut microbiota for each strain/feeding status from the single time point bacterial load analysis presented in Fig 1. (DOCX)

S5 Table Zero-inflated data analysis for multiple time point bacterial load analysis shown in Fig 2 to assess the effect of strain, feeding status and time post blood meal (tpbm) on midgut microbial load. (DOCX)

S1 Data Sequences for 16S profiling in FASTA format. Sample names for each treatment are as follows: Rockefeller strain sucrose = GD.SMS.RockSuc.mg.Mar3.Pooled, Rockefeller strain blood fed = GD.SMS.RockBlood.mg.Mar3.Pooled, Singapore strain sucrose: = GD.SMS.SingSuc.mg.Mar3.Pooled, Singapore strain blood fed = GD.SMS.SingBlood.mg.Mar3.Pooled.
(ZIP)

S2 Data Sequence identifier data for each FASTA sequence. For each sequence ID the following is provided: Organism, clone, collection date, location, host, isolation source, and BioSample number.
(ZIP)
