# Supplementary figures and images for: Amino acid metabolic signaling influences Aedes aegypti midgut microbiome variability
Source: PLoS Negl Trop Dis. 2017 Jul 28;11(7):e0005677. doi: 10.1371/journal.pntd.0005677 (PMC5549995; doi:10.1371/journal.pntd.0005677)

**A**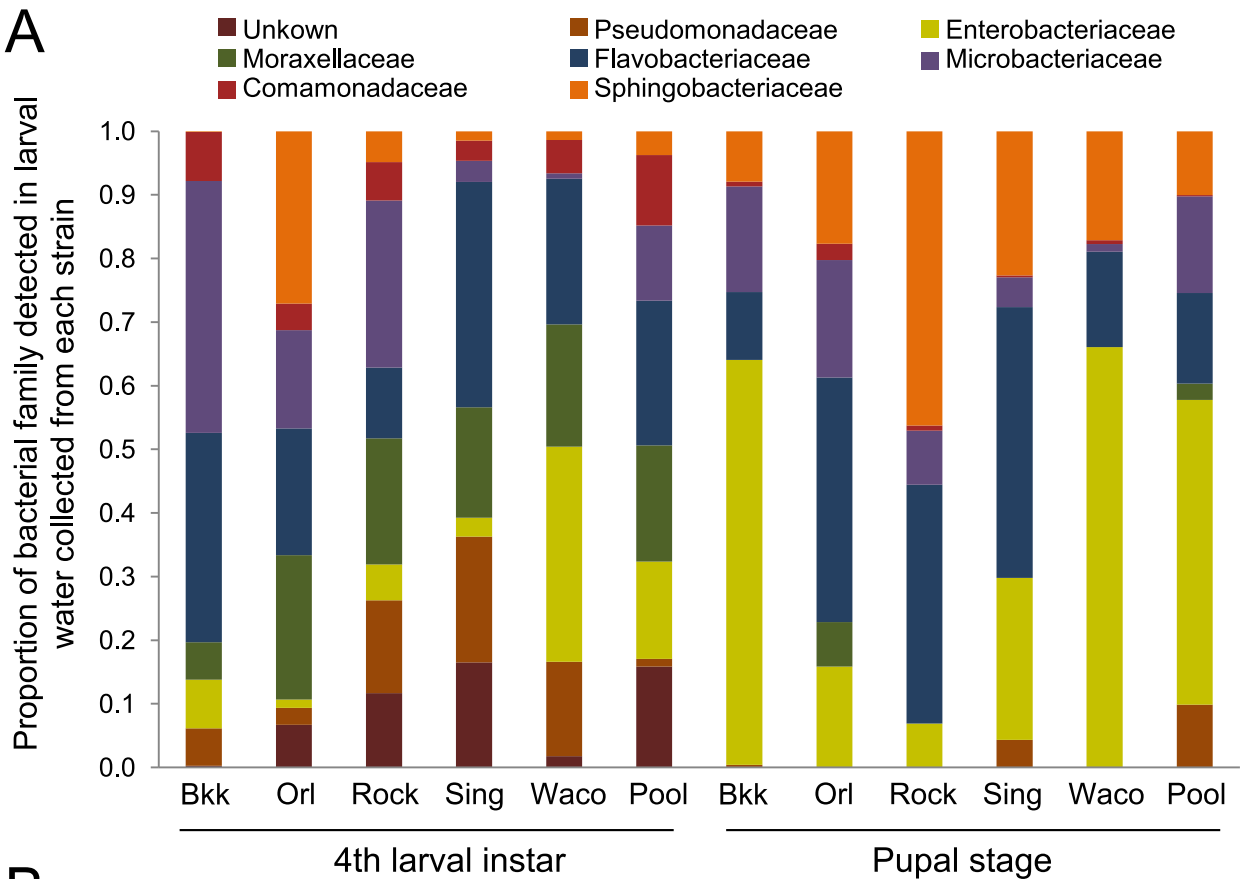**B**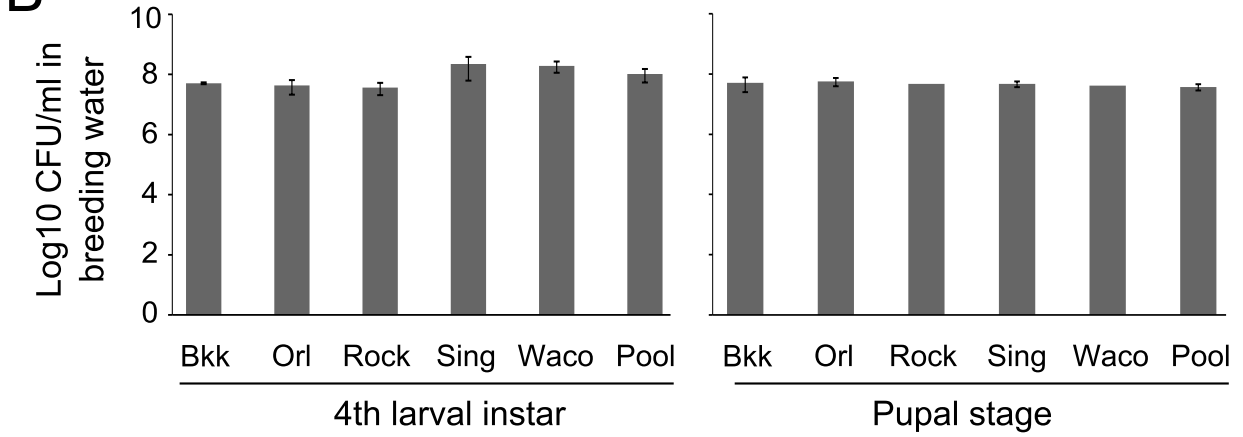

Supplement: S1 Fig — We sampled an aliquot of larval water from each strain at the 4th instar and pupal stages of development. We also sampled an aliquot from the rearing water after it had been pooled across all strains at each stage. Pooled water was reallocated to rearing trays or pupal cups for each strain to increase similarity in bacterial environment between strains. Each sample was serially diluted and 50μL of each dilution was spread on LB agar and grown at room temperature for 48 hours. Colony types were then characterized by form, margin, elevation, color and translucency and each type was quantified for each sample. To identify each colony type, the 16S rDNA gene was sequenced and compared to existing 16S sequences via the Ribosomal Database Project. Each strain was sampled over two replicate experiments with the exception of Rockefeller and Waco pupal stage, for which samples were only successfully analyzed from a single replicate. (A) Composition of LB-cultivable bacteria detected in larval/pupal rearing water. 4th instar larval breeding water contained eight bacterial families, all of which were detected in all strains, with the exception of the Bangkok strain, for which six of the eight bacterial families were detected. Pupal rearing water had five of seven bacterial families detected in every strain. (B) Total number of LB-cultivable bacteria in rearing water At the L4 stage, average total CFU/ml rearing water ranged from 3.6 × 10^7^ to 2.2 × 10^8^ and total CFU/ml did not differ significantly between rearing water of the strains (pstrain = 0.1991). At the pupal stage, average CFU/ml rearing water ranged from 4.09×10^7^ to 5.66 × 10^7^, and again CFU/ml did not differ significantly between the rearing water of the strains (S1 Fig, pstrain = 0.9928). Bkk: Bangkok, Orl: Orlando, Rock: Rockefeller, Sing: Singapore. (PDF) [file pntd.0005677.s001.pdf]

## Rockefeller

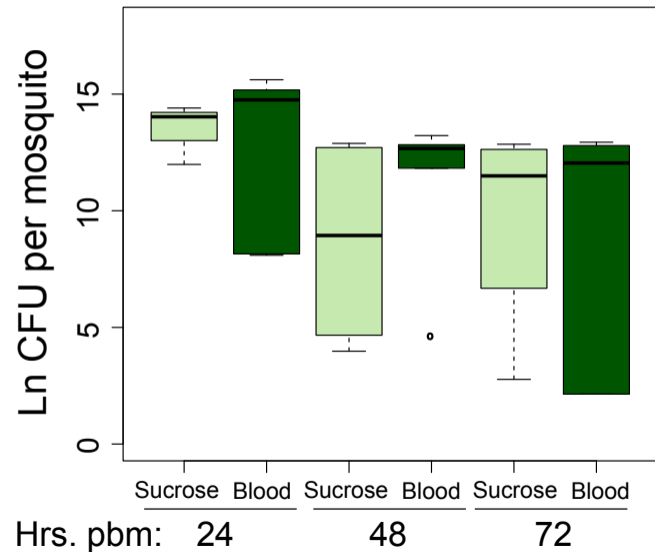

## Singapore

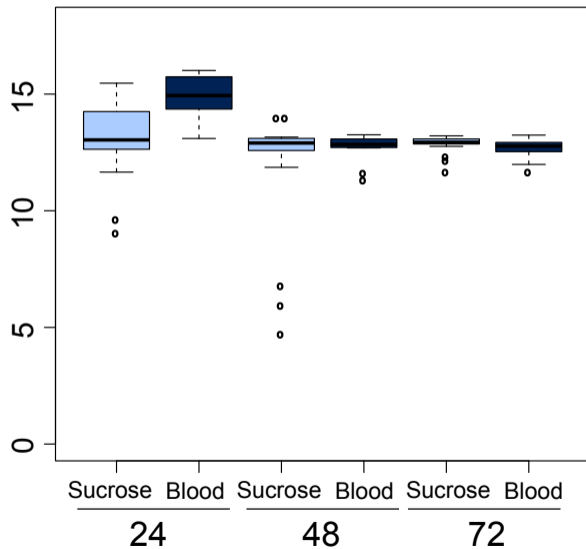

Supplement: S3 Fig — We quantified E. meningoseptica colonies from females sampled in the multiple time point bacterial load analysis shown in Fig 2 and assessed the effect of strain and feeding status at each time point using a general linear model. At 24 hours post blood meal (pbm), we detected a significant effect of feeding (p = 4.87 x 10^−5^) but failed to detect a significant effect of strain (p = 0.513). At 48 hours pbm, neither feeding status (p = 0.967) nor strain (p = 0.069) were significant. At 72 hours pbm, strain was highly significant (p = 0.0001) while feeding status was not (p = 0.232). (PDF) [file pntd.0005677.s003.pdf]

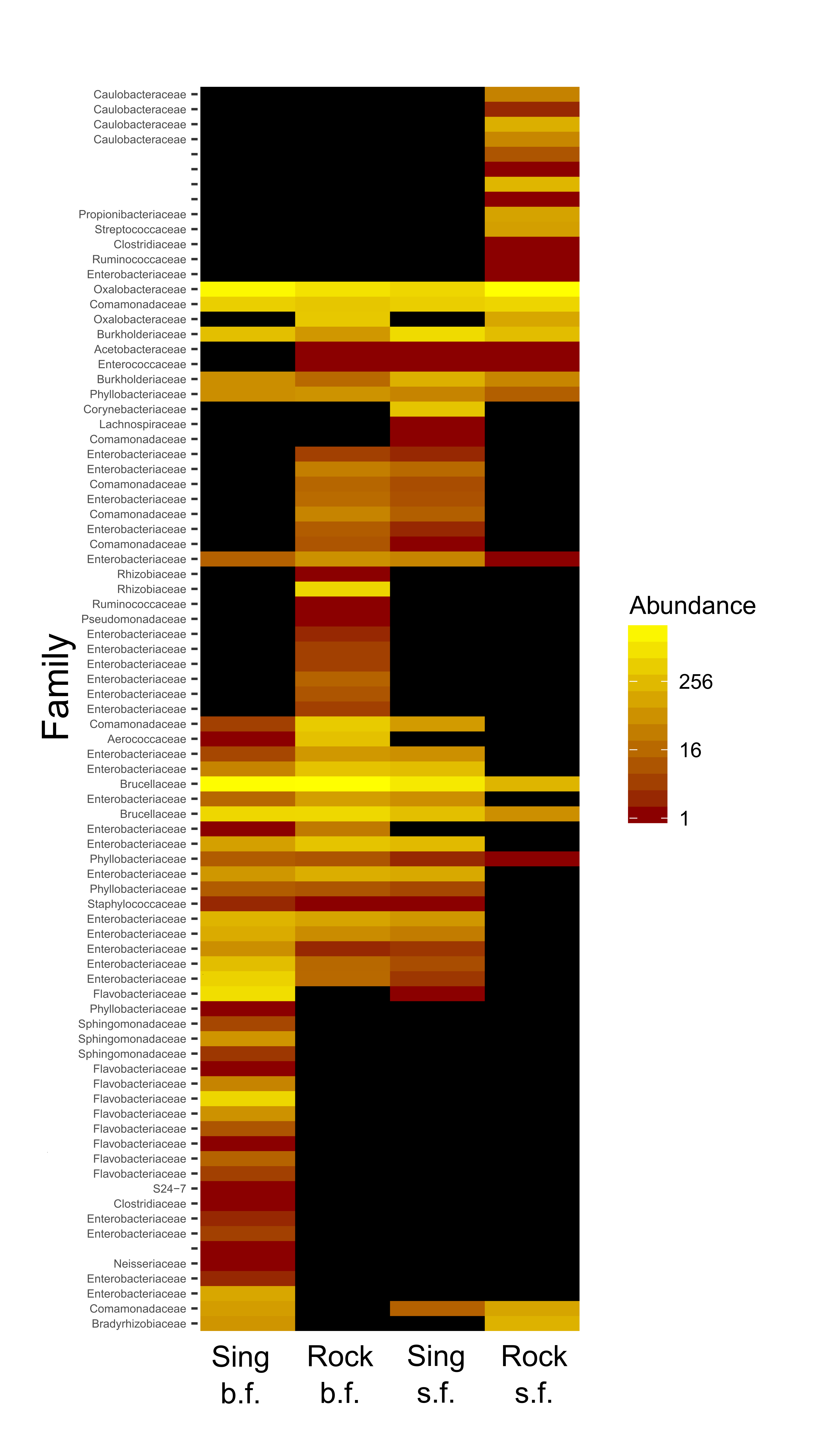

Supplement: S4 Fig — Each row represents abundance of reads from each OTU and is labeled by family. We chose to present the data at family level because many of the OTUs could not be assigned to a genus with high confidence. (TIF) [file pntd.0005677.s004.tif]

### AAEL003125

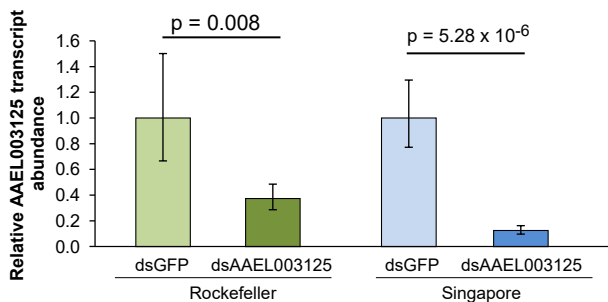

### AAEL004137

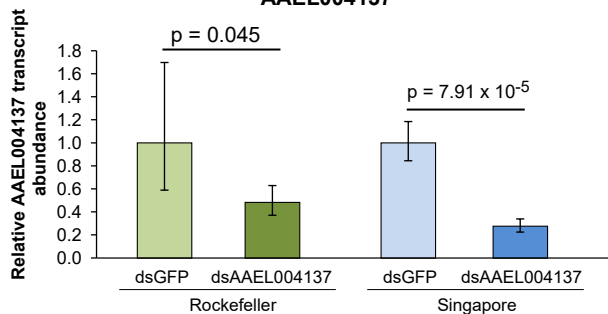

### AAEL006928

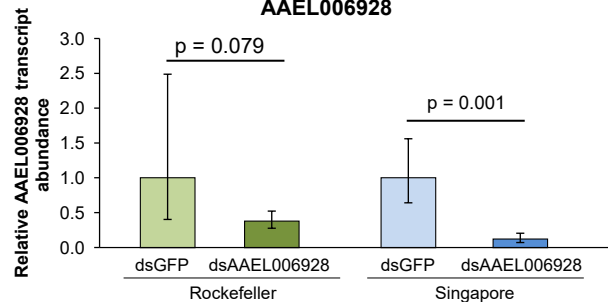

Supplement: S5 Fig — We injected mosquitoes with 200ng dsRNA targeting one of the candidate genes or eGFP as a control and dissected pools of 8 midguts 2 days post injection. We then extracted RNA from each pool and performed qPCR to quantify the levels of each gene as well as a reference gene, S7. We assessed differences between treatment and control samples by Student’s t-test, and we used the delta delta CT method to calculate relative expression levels, where silenced samples are shown relative to the eGFP control within each strain (i.e. Rockefeller and Singapore eGFP are both standardized to 1). (PDF) [file pntd.0005677.s005.pdf]

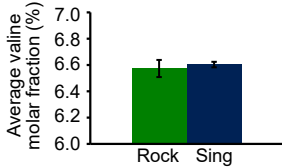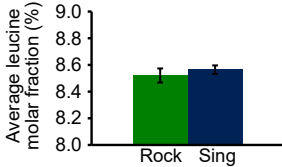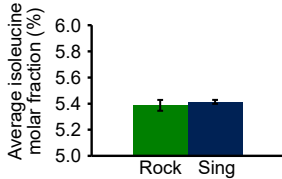

Supplement: S6 Fig — We dissected midgut tissue in pools of 25 from Rockefeller and Singapore female mosquitoes 5–7 days post eclosion and analyzed amino acid composition via ion-exchange chromatography and subsequent ninhydrin reaction detection. Percent molar fraction refers to the percent of total amino acid (nmoles) that was identified to be valine, leucine, or isoleucine in each sample. The experiment was repeated three independent times, and error bars represent one standard error. (PDF) [file pntd.0005677.s006.pdf]

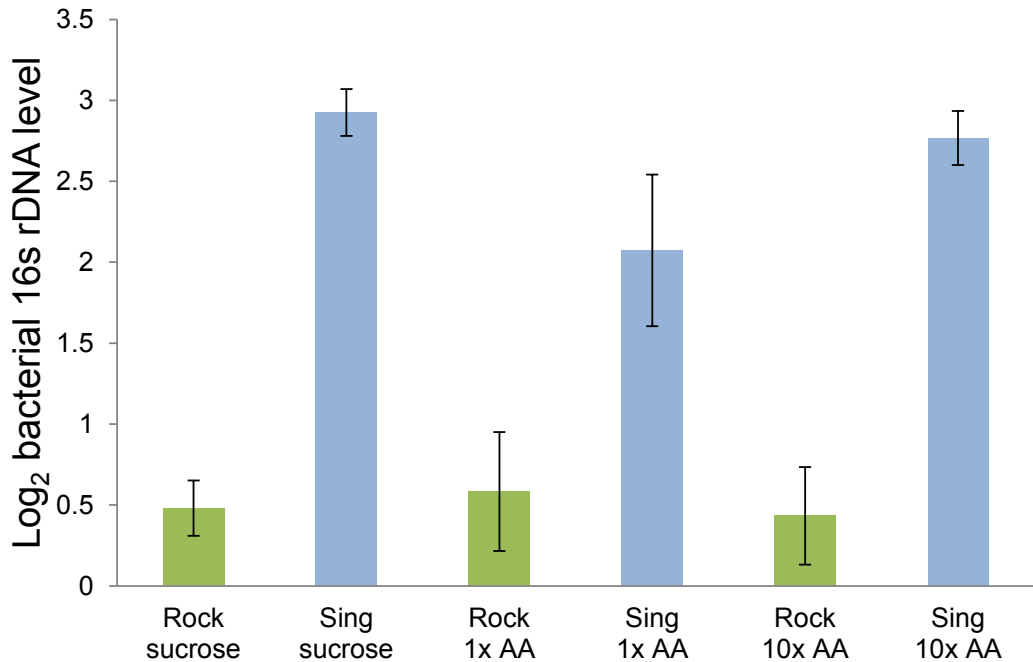

Supplement: S7 Fig — We reared Rockefeller and Singapore mosquitoes in parallel, and at 3–5 days post-eclosion, females from each strain were given a meal of either: 3% sucrose, 3% sucrose + 1X AA, or 3% sucrose + 10X AA, where 1X AA = 0.00034g Ile + 0.00069g Val + 0.00069g Leu per 100ml 3% sucrose. After two days, we dissected one pool of eight midguts for each strain/treatment combination. This entire experiment was repeated four independent times. We extracted DNA from each midgut pool and performed qPCR to quantify levels of the bacterial16S rDNA gene and A. aegypti S7 reference gene. Y-axis values are average inverse delta CT values, i.e. -1*(CT16S –CTS7) for each treatment. Because CT values are Log2, a difference of 1 on the y-axis corresponds to a 2-fold change in 16S DNA abundance. We performed an ANOVA to test the effect of strain, feeding treatment, and an interaction between these factors. Strain was highly significant (p = 7.78 x 10^−8^), but we did not detect an effect of feeding treatment (p = 0.440) nor a two-way interaction (p = 0.237). (PDF) [file pntd.0005677.s007.pdf]

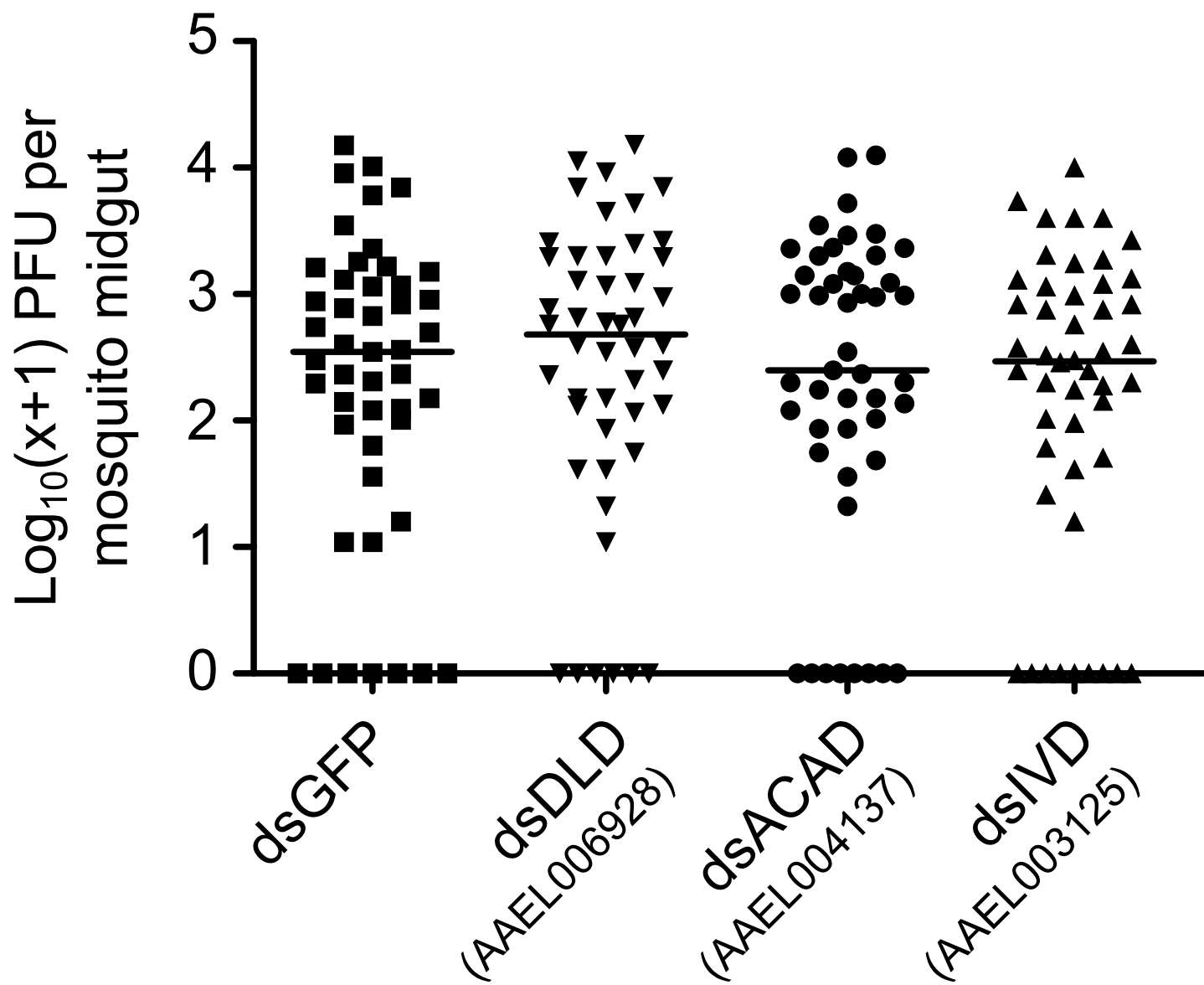

Supplement: S8 Fig — Rockefeller strain females were injected with dsRNA targeting three genes in the branched chain amino acid degradation pathway (AAEL006928, AAEL004137, AAEL003125) or eGFP as a control. Forty-eight hours after dsRNA injection, females were orally infected with dengue virus via infectious blood meal. Seven days after blood feeding, midguts were dissected from females from each treatment, and viral titer was assayed for each individual by plaque assay. Analysis by one-way ANOVA revealed no significant effect of treatment relative to GFP controls. The entire experiment was replicated twice and sample sizes for each treatment are as follows: ndsGFP = 47, ndsAAEL006928 = 48, ndsAAEL004137 = 47, ndsAAEL003125 = 48. DLD = dihydrolipoamide dehydrogenase, ACAD = acyl-CoA dehydrogenase, IVD = isovaleryl-CoA dehydrogenase. (PDF) [file pntd.0005677.s008.pdf]
