# Supplementary material for: Amino acid metabolic signaling influences Aedes aegypti midgut microbiome variability
Source: PLoS Negl Trop Dis. 2017 Jul 28;11(7):e0005677. doi: 10.1371/journal.pntd.0005677 (PMC5549995; doi:10.1371/journal.pntd.0005677)
Supplement: S2 Fig — These data were taken from the same samples used in the single time point bacterial load analysis presented in Fig 1. We identified each colony type using 16S rRNA gene sequencing and combined counts at the family level post hoc within each treatment group to obtain an overall percentage. (PDF) [file pntd.0005677.s002.pdf]

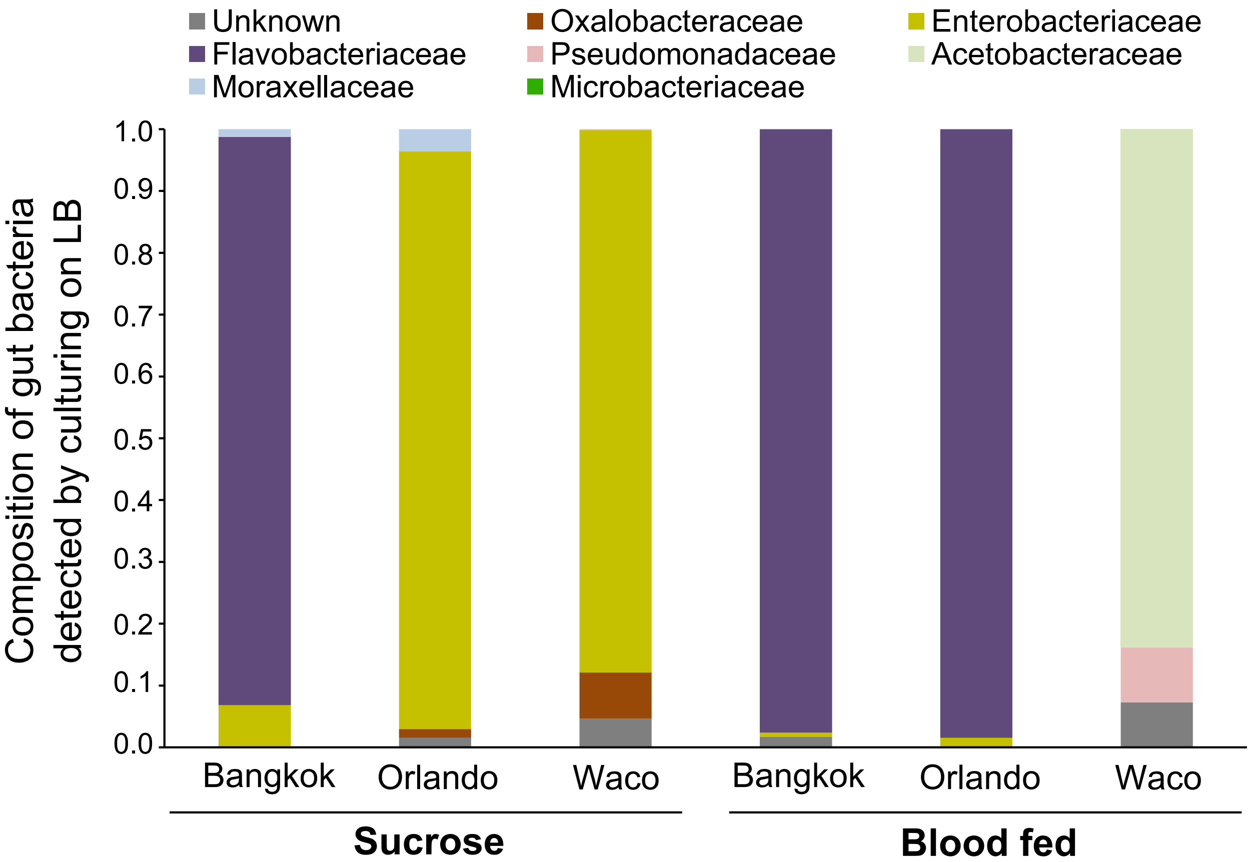

Unknown
Oxalobacteraceae
Enterobacteriaceae
Flavobacteriaceae
Pseudomonadaceae
Acetobacteraceae
Moraxellaceae
Microbacteriaceae
Composition of gut bacteria detected by culturing on LB
1.0
0.9
0.8
0.7
0.6
0.5
0.4
0.3
0.2
0.1
0.0
Bangkok
Orlando
Waco
Bangkok
Orlando
Waco
Sucrose
Blood fed
