# Supplementary material for: Amino acid metabolic signaling influences Aedes aegypti midgut microbiome variability
Source: PLoS Negl Trop Dis. 2017 Jul 28;11(7):e0005677. doi: 10.1371/journal.pntd.0005677 (PMC5549995; doi:10.1371/journal.pntd.0005677)
Supplement: S1 Table — (DOCX) [file pntd.0005677.s010.docx]

**S1 Table. Primers used for qPCR, and dsRNA synthesis.**

| Primer name | Sequence | Use |
| --- | --- | --- |
| 16s-F | TCCTACGGGAGGCAGCAGT | 16s qPCR |
| 16s-R | GGACTACCAGGGTATCTAATCCTGTT | 16s qPCR |
| qS7-gDNA-F2 | TAGACACCCTGAAGTTGTTGCAAAT | S7 qPCR - gDNA |
| qS7-gDNA-R2 | TGTATATGCGCATTAGTCTCATCAA | S7 qPCR - gDNA |
| qAe-S7-F | GCAGACCACCATTGAACACA | S7 qPCR - cDNA |
| qAe-S7-R | CACGTCCGGTCAGCTTCTTG | S7 qPCR - cDNA |
| T7-EGFP-F | CGACGATAATACGACTCACTATAGGGTTCATCTGCACCACCGGC | eGFP dsRNA synthesis |
| T7-EGFP-R | CGACGATAATACGACTCACTATAGGGCTGGTAGTGGTCGGCGAG | eGFP dsRNA synthesis |
| T7-AAEL003125-F128 | TAATACGACTCACTATAGGGCTGCGACCTCAGGGAAGTG | AAEL003125 dsRNA synthesis |
| T7-AAEL003125-R505 | TAATACGACTCACTATAGGGGTTTCGGTGGATTTGGTTGA | AAEL003125 dsRNA synthesis |
| T7-AAEL004137-F220 | TAATACGACTCACTATAGGGCGACTTCGCCGATAATGAG | AAEL004137 dsRNA synthesis |
| T7-AAEL004137-R573 | TAATACGACTCACTATAGGGCCTGCATCCGATCCATTAC | AAEL004137 dsRNA synthesis |
| T7-AAEL006928-F1088 | TAATACGACTCACTATAGGGGGCCGTCGTCCATACACT | AAEL006928 dsRNA synthesis |
| T7-AAEL006928-R1451 | TAATACGACTCACTATAGGGTGTCGTTATTGGTCTTGGCA | AAEL006928 dsRNA synthesis |
| qAAEL003125F | CTGCGATGTAGCCTTCGAGT | AAEL003125 qPCR |
| qAAEL003125R | GATAAGCACGGCAAGCGTTC | AAEL003125 qPCR |
| qAAEL004137F | CGCTCAGGCATCTCTCGAAT | AAEL004137 qPCR |
| qAAEL004137R | CGGCGATCTTTTGCTGGATG | AAEL004137 qPCR |
| qAAEL006928F | ACAATGTCCGCCTGGATCTG | AAEL006928 qPCR |
| qAAEL006928R | GTGTTCGGTCCGGTAATGGT | AAEL006928 qPCR |
