# Supplementary material for: Amino acid metabolic signaling influences Aedes aegypti midgut microbiome variability
Source: PLoS Negl Trop Dis. 2017 Jul 28;11(7):e0005677. doi: 10.1371/journal.pntd.0005677 (PMC5549995; doi:10.1371/journal.pntd.0005677)
Supplement: S2 Table — (DOCX) [file pntd.0005677.s011.docx]

**S2 Table. Bacteria used in blood meal for genome-wide gene expression experiment.**

| Bacteria | Source |
| --- | --- |
| *Comamonas testosteroni* | Panama, field mosquito^1^ |
| *Pantoea dispersa* | Panama, field mosquito^1^ |
| *Lactococcus lactis* | Panama, field mosquito^1^ |
| *Asaia borgogensis* | Panama, field mosquito^1^ |
| *Pseudomonas putida* | Maryland, USA, laboratory mosquito (this study). |
| *Acinetobacter* | Maryland, USA, laboratory mosquito (this study). |
| *Elizabethkingia meningoseptica* | Maryland, USA, laboratory mosquito (this study). |

1. Ramirez *et al.* 2012
