# Supplementary material for: Amino acid metabolic signaling influences Aedes aegypti midgut microbiome variability
Source: PLoS Negl Trop Dis. 2017 Jul 28;11(7):e0005677. doi: 10.1371/journal.pntd.0005677 (PMC5549995; doi:10.1371/journal.pntd.0005677)
Supplement: S3 Table — (DOCX) [file pntd.0005677.s012.docx]

| **S3 Table. Zero-inflated data analysis for single time point bacterial load analysis assays presented in Figure 1 to assess the effect of strain and feeding status on midgut microbial load.** | | | | |
| --- | --- | --- | --- | --- |
|  | Dropped term | d.f. | Chi sq. | p value |
|  | None | 21 |  |  |
| Count model: | Strain x feeding | 17 | 5.25 | 0.2625 |
|  | feeding | 16 | 0.7082 | 0.4000 |
|  | Strain | 12 | 67.935 | 6.191 × 10^-14^ |
| Presence/absence model: | Strain x feeding | 17 | 11.994 | 0.0174 |
