# Supplementary material for: Amino acid metabolic signaling influences Aedes aegypti midgut microbiome variability
Source: PLoS Negl Trop Dis. 2017 Jul 28;11(7):e0005677. doi: 10.1371/journal.pntd.0005677 (PMC5549995; doi:10.1371/journal.pntd.0005677)
Supplement: S4 Table — (DOCX) [file pntd.0005677.s013.docx]

**S4 Table. Prevalence of LB-cultivable midgut microbiota for each strain/feeding status from the single time point bacterial load analysis presented in Figure 1.**

|  | % of individuals with > 0 CFU  (n/total) | % of individuals with 0 CFU  (n/total) |
| --- | --- | --- |
| Bkk Sucrose | 92.3% (12/13) | 7.7% (1/13) |
| Orl Sucrose | 72.2% (13/18) | 27.7% (5/18) |
| Rock Sucrose | 94.4% (17/18) | 5.5% (1/18) |
| Sing Sucrose | 100% (13/13) | 0% (0/13) |
| Waco Sucrose | 57.1% (4/7) | 42.9% (3/7) |
| Bkk blood fed | 18.2% (2/11) | 81.8% (9/11) |
| Orl blood fed | 29.2% (7/24) | 70.8% (17/24) |
| Rock blood fed | 20% (5/25) | 80% (20/25) |
| Sing blood fed | 90.1% (10/11) | 0.9% (1/11) |
| Waco blood fed | 62.5% (5/8) | 37.5% (3/8) |
