# Supplementary material for: Amino acid metabolic signaling influences Aedes aegypti midgut microbiome variability
Source: PLoS Negl Trop Dis. 2017 Jul 28;11(7):e0005677. doi: 10.1371/journal.pntd.0005677 (PMC5549995; doi:10.1371/journal.pntd.0005677)
Supplement: S5 Table — (DOCX) [file pntd.0005677.s014.docx]

| **S5 Table. Zero-inflated data analysis for multiple time point bacterial load analysis shown in** **Figure 2 to assess the effect of strain, feeding status and time post blood meal (tpbm) on midgut microbial load.** | | | | |
| --- | --- | --- | --- | --- |
|  | Dropped term | d.f. | Chi sq. | p value |
|  | None | 25 |  |  |
| Count model: | strain x feeding x tpbm | 23 | 1.6009 | 0.4491 |
|  | strain x feeding | 22 | 0.0589 | 0.8082 |
|  | strain x tpbm | 20 | 0.4643 | 0.7928 |
|  | feeding x tpbm | 18 | 10.238 | 0.0060 |
|  | strain | 19 | 5.1172 | 0.0237 |
| Presence/absence model: | strain x feeding x tpbm | 18 | 0.6477 | 0.7233 |
|  | strain x feeding | 17 | 4.2603 | 0.0390 |
|  | strain x tpbm | 16 | 0.3336 | 0.8464 |
|  | feeding x tpbm | 14 | 1.532 | 0.4649 |
|  | tpbm | 12 | 0.7148 | 0.6995 |
